# CDK4 T172 Phosphorylation Is Central in a CDK7-Dependent Bidirectional CDK4/CDK2 Interplay Mediated by p21 Phosphorylation at the Restriction Point

**DOI:** 10.1371/journal.pgen.1003546

**Published:** 2013-05-30

**Authors:** Xavier Bisteau, Sabine Paternot, Bianca Colleoni, Karin Ecker, Katia Coulonval, Philippe De Groote, Wim Declercq, Ludger Hengst, Pierre P. Roger

**Affiliations:** 1 WELBIO and Institute of Interdisciplinary Research (IRIBHM), Université Libre de Bruxelles, Brussels, Belgium; 2 Division of Medical Biochemistry, Biocenter, Innsbruck Medical University, Innsbruck, Austria; 3 Department for Molecular Biomedical Research, VIB, and Department of Biomedical Molecular Biology, Ghent University, Ghent, Belgium; Fred Hutchinson Cancer Research Center, United States of America

## Abstract

Cell cycle progression, including genome duplication, is orchestrated by cyclin-dependent kinases (CDKs). CDK activation depends on phosphorylation of their T-loop by a CDK–activating kinase (CAK). In animals, the only known CAK for CDK2 and CDK1 is cyclin H-CDK7, which is constitutively active. Therefore, the critical activation step is dephosphorylation of inhibitory sites by Cdc25 phosphatases rather than unrestricted T-loop phosphorylation. Homologous CDK4 and CDK6 bound to cyclins D are master integrators of mitogenic/oncogenic signaling cascades by initiating the inactivation of the central oncosuppressor pRb and cell cycle commitment at the restriction point. Unlike the situation in CDK1 and CDK2 cyclin complexes, and in contrast to the weak but constitutive T177 phosphorylation of CDK6, we have identified the T-loop phosphorylation at T172 as the highly regulated step determining CDK4 activity. Whether both CDK4 and CDK6 phosphorylations are catalyzed by CDK7 remains unclear. To answer this question, we took a chemical-genetics approach by using analogue-sensitive CDK7(as/as) mutant HCT116 cells, in which CDK7 can be specifically inhibited by bulky adenine analogs. Intriguingly, CDK7 inhibition prevented activating phosphorylations of CDK4/6, but for CDK4 this was at least partly dependent on its binding to p21*^cip1^*. In response to CDK7 inhibition, p21-binding to CDK4 increased concomitantly with disappearance of the most abundant phosphorylation of p21, which we localized at S130 and found to be catalyzed by both CDK4 and CDK2. The S130A mutation of p21 prevented the activating CDK4 phosphorylation, and inhibition of CDK4/6 and CDK2 impaired phosphorylations of both p21 and p21-bound CDK4. Therefore, specific CDK7 inhibition revealed the following: a crucial but partly indirect CDK7 involvement in phosphorylation/activation of CDK4 and CDK6; existence of CDK4-activating kinase(s) other than CDK7; and novel CDK7-dependent positive feedbacks mediated by p21 phosphorylation by CDK4 and CDK2 to sustain CDK4 activation, pRb inactivation, and restriction point passage.

## Author Summary

In the cell cycle, duplication of all the cellular components and subsequent cell division are governed by a family of protein kinases associated with cyclins (CDKs). Related CDK4 and CDK6 bound to cyclins D are the first CDKs to be activated in response to cell proliferation signals. They thus play a central role in the cell multiplication decision, especially in most cancer cells in which CDK4 activity is highly deregulated. We have identified the activating T172 phosphorylation instead of cyclin D expression as the highly regulated step determining CDK4 activation. This finding contrasts with the prevalent view that the only identified metazoan CDK-activating kinase, CDK7, is constitutively active. By using human cells genetically engineered for specific chemical inhibition of CDK7, we found that CDK7 activity was indeed required for CDK4 activation. However, this dependence was conditioned by CDK4 binding to the CDK inhibitory protein p21, which increased in response to CDK7 inhibition. Further investigation revealed that CDK7 inhibition affects a major phosphorylation of p21, which we found to be required for CDK4 activation and performed by CDK4 itself and CDK2. Thus, depending on CDK7 activity, CDK4 and CDK2 facilitate CDK4 activation, generating novel positive feedbacks involved in the cell cycle decision.

## Introduction

The cell cycle commitment at the restriction (R) point in G1 phase is initiated by inactivating phosphorylations of the central cell cycle/tumor suppressor pRb by CDK4 and CDK6, which are activated by D-type cyclins induced by mitogenic/oncogenic signaling [1]–[3]. pRb phosphorylation is then maintained independently of cyclins D, and hence of mitogens, by a positive feedback loop linking pRb to E2F-dependent transcription of cyclin E, which leads to CDK2 activation and further phosphorylation of pRb [4]. This, together with other positive-feedback circuits, such as E2F inducing its own transcription and the mutual inhibition between cyclin E-CDK2 and p27^Kip1^, has been shown to generate a bistable pRb-E2F switch to convert graded mitogen inputs into all-or-none E2F responses and thus cell cycle commitment [5]. The effects of the Cip/Kip CDK inhibitors p21 and p27 in this process are complex and remain much debated [6], [7]. p21 is the main transcriptional target involved in replicative senescence and p53-dependent cell cycle inhibition in response to DNA damage. But in unperturbed cell cycles, it is also transiently induced by mitogenic stimuli during G1, before its required disappearance at the G1/S transition [8]. While p21 inhibits CDK2 [9], it also plays essential positive roles by stabilizing cyclin D-CDK4 complexes and allowing their nuclear import [3], [10]. How can p21 and p27 bind to CDK4 complexes and either inhibit or not inhibit their activity is poorly understood [3], [6], [8]. One debated possibility is related to different stoichiometries of the binding of these proteins to cyclin-CDK complexes [10], [11]. On the other hand, as first exemplified by T187 phosphorylation of p27 [12], phosphorylations of Cip/Kip proteins, including by oncogenic tyrosine kinases, have also emerged as other potential mechanisms for CDK regulation [13]–[17].

CDK4/6 activity is deregulated through various mechanisms in many human tumors [18], [19]. Such deregulation is crucial for various oncogenic transformation processes [20]–[22] suggesting that many cancer cells are addicted to high CDK4/6 activity [19], [23]–[25]. A CDK4/6 inhibitor (PD0332991 [24]–[29]) that effectively halts tumor growth in preclinical models and in patients is being tested in phase II clinical trials on various pRb-proficient chemotherapy-resistant cancers.

Activation of CDK4/6 is a still poorly understood multistep process that includes an activating phosphorylation in the T-loop at T172 in CDK4 [30], [31] and at T177 in CDK6 [32], [33]. However, these crucial phosphorylations have been barely studied because easy detection tools are unavailable. Despite constitutive cyclin D expression, defective expression of INK4 CDK4/6 inhibitory proteins and frequent overexpression of cyclin E [34], many tumor cells maintain at least partial regulation at the R point. By using 2D-gel electrophoresis to separate the phosphorylated forms of CDK4 and CDK6 bound to their regulatory partners, we have identified the activating T172 phosphorylation as the last, separately regulated, critical step of CDK4 activation in such tumor cells [3], [35]–[37] and in various normal cells [31], [38], [39]. Importantly, our investigation did not confirm a significant presence of the inhibitory phosphorylation of CDK4 at the Y17 site (or CDK6 at Y25) [31], [33], which corresponds to crucial Y15/T14 phosphorylations of CDK2 and CDK1 [40], [41].

This determining regulation of CDK4 T172 phosphorylation contrasts with the prevalent concept that the activating phosphorylations of the different CDKs, including CDK4 and CDK6, are performed solely by the only CDK-activating kinase (CAK) known in metazoan cells, which is composed of the cyclin H-CDK7-Mat1 complex [42]–[44]. This complex is constitutively active [31], [45], even when assayed on CDK4 [36], [37], [46]. Other puzzling observations also raised doubts that CAK/CDK7 could be the main (or the sole) CDK4-activating kinase (reviewed in [35]). For instance, CDK4 phosphorylation can be differentially regulated in cyclin D1 or cyclin D3 complexes [36], [47]. Moreover, T172-phosphorylated CDK4 is enriched in p27-bound cyclin D-CDK4 complexes [31], whereas p27 binding prevents CDK phosphorylation by CAK/CDK7 [15], [48]. Finally, the regulation of CDK4 phosphorylation does not apply to CDK6, which remains barely phosphorylated in many cells [31], [33]. We ascribed this to the absence of a conserved adjacent proline residue that is uniquely present in the phosphoacceptor domain of CDK4 (QMAL**T** PVVVT in CDK4 *vs* QMAL**T**SVVVT in CDK6) [30], [33]. P173 mutations of CDK4 abrogated its T172 phosphorylation in cells, while S178P mutation of CDK6 led to its complete T177 phosphorylation [33]. However, CDK2 and CDK6 are much better *in vitro* substrates of CAK/CDK7 than CDK4 [31]–[33], [49]. Moreover, P173S mutation of CDK4 did not impair its *in vitro* activation by CAK [33], which is consistent with the concept that CDK recognition by CAK does not depend on a consensus sequence around the phosphoacceptor site [49]–[52].

We thus hypothesized that unlike CDK2 and CDK1 [53], CDK4 is not activated in cells by cyclin H-CDK7, but by one or several proline-directed kinase(s). The hypothesis that animal cells have multiple CAKs like yeasts and plant cells [44], [54] is not novel [44], [55], [56] and it could help to resolve the complex issue of the divergent constrains of the dual roles of CDK7 in cell cycle and mRNA transcription [50]. Alternatively, CDK7 could still be the catalytic subunit of the proline-directed CDK4-activating kinase that we postulated. Indeed, cyclin H-CDK7-Mat1 associated with TFIIH phosphorylates non-CDK substrates at T/S-P motifs [49].

RNAi-mediated (partial) depletion of CDK7 is generally insufficient to affect cell cycle progression and thus CDK activity. Moreover, approaches based on inhibition of CDKs or their knockout (or knockdown) have generated divergent conclusions [57]. As no specific inhibitor of CDK7 has been developed, Robert Fisher's group has replaced in HCT116 human colon carcinoma cells the two CDK7 alleles by a mutated CDK7 (F91G) that can be specifically inhibited by “bulky” adenine analogs (K7AS HCT116 cells) [53]. These cells enabled us to (i) demonstrate crucial –but unexpectedly complex and partly indirect– involvements of CDK7 in CDK4 and CDK6 activation, (ii) uncover novel positive feedback pathways mediated by p21 phosphorylation and involving CDK7-dependent activities of CDK4 and CDK2 in CDK4 activation, and (iii) demonstrate the existence of non-CDK7 CDK4-activating kinase(s).

## Results

### Acute requirement for CDK7 activity in CDK4 and CDK6 activation in HCT116 cells

Cell cycle progression and kinetics of CDK4 phosphorylation upon stimulation of serum-deprived K7AS HCT116 cells with 10% serum were analyzed as detailed in Figure S1 and its legend. The relative presence of phosphorylated and non-phosphorylated CDK4 forms in coimmunoprecipitated complexes was assessed by 2D-gel electrophoresis as previously [31] (Figure S1C). We have previously identified the most negatively charged form as the T172-phosphorylated CDK4 using several approaches: [32P]phosphate incorporation, a phospho(T172)-specific CDK4 antibody, *in vitro* phosphorylation by recombinant CAK, and analysis of T172A-mutated CDK4 [31], [33]. Here, the phosphorylation of cyclin D1-bound CDK4 appeared at 2–3 h into G1 phase, whereas the phosphorylation of cyclin D3-bound CDK4 was detectable in serum-deprived cells and increased much later at 12 h and subsequent time points, when most cells were in S–G2 phases (Figure S1C).

**Figure 1:**
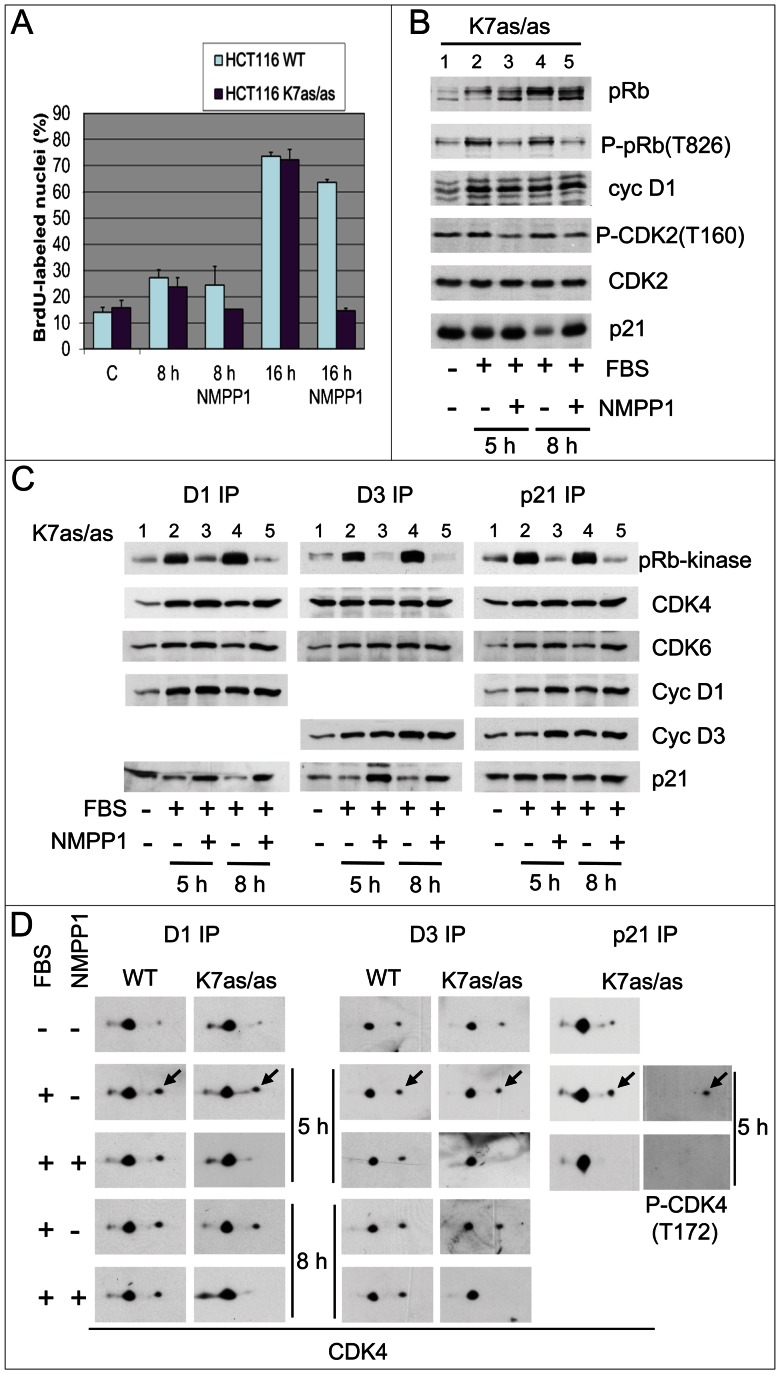
Specific inhibition of CDK7 by 1-NMPP1. Specific inhibition of CDK7 by 1-NMPP1 prevents DNA synthesis (A), pRb phosphorylation (B), CDK4/6 pRb-kinase activity (C) and CDK4 activating phosphorylation (D). HCT116 WT or K7AS (K7as/as) cells were stimulated (+) or not stimulated (−) with FBS for the indicated times in the absence (−) or presence (+) of 1-NMPP1. (A) DNA synthesis was evaluated from duplicate dishes by counting the percentage of cells having incorporated BrdU during the last 30 min of stimulation. (B) Western blotting analysis with the indicated antibodies from whole-cell lysates. (C) Cell lysates were immunoprecipitated (IP) with anti-cyclin D1 (D1), anti-cyclin D3 (D3) or anti-p21 (p21) antibodies and were assayed for their pRb-kinase activity, separated by SDS-PAGE and immunoblotted with the indicated antibodies (C). (D) The same immunoprecipitates were separated by 2D gel electrophoresis followed by immunodetection using antibodies directed against CDK4 or T172-phosphorylated CDK4. Arrows, T172-phosphorylated form of CDK4.

To test whether CDK7 inhibition affects the activation of CDK4 through T172-phosphorylation, serum-deprived wild-type (wt) and K7AS HCT116 cells were re-stimulated by serum in the continuous presence or absence of the bulky adenine analog 1-NMPP1 (10 µM) to specifically inhibit CDK7 activity. As previously shown [53], 1-NMPP1 prevented the induction of DNA synthesis in K7AS but not in wt HCT116 cells (Figure 1A). This was associated with a similar inhibition of the stimulated T826 phosphorylation of pRb in K7AS but not in wt cells, without any inhibition of the accumulation of cyclin D1, cyclin D3 or CDK4 (Figure 1B; Figure S2A). Instead, cyclin D1 accumulation increased further at 8 and 16 h in response to CDK7 inhibition in K7AS cells (Figure S2A), likely due to impairment of the degradation it undergoes during S-phase progression. Serum-stimulated T160 phosphorylation of CDK2 was also inhibited only in K7AS cells, as previously demonstrated [53]. Interestingly, a marked accumulation of p21 in response to CDK7 inhibition was observed at 8 h and even more at 16 h (Figure 1B; Figure S2A).

In the same experiments, we analyzed in K7AS cells the effect of CDK7 inhibition on the pRb-kinase activity of CDK4 and CDK6, both of which were immunoprecipitated using antibodies against cyclin D1, D3, p21 or CDK6. The protein composition of these complexes was also analyzed by western blotting (Figure 1C; Figure S2C). CDK7 inhibition by 1-NMPP1 largely or totally inhibited the serum-stimulated activity of CDK4 and CDK6 without reducing their association with cyclin D1 or cyclin D3. As generally observed in unperturbed cell cycles [3], [10], [58], p21-bound CDK4/6 was largely active in response to serum stimulation, and here again its activity was inhibited by 1-NMPP1 treatment (Figure 1C).

The inhibition of pRb-kinase activity in response to selective CDK7 inhibition was associated with reduced phosphorylations of CDK4 and/or CDK6. Co-immunoprecipitation of these kinases and separation of their phosphorylated forms by 2D-gel electrophoresis showed that continuous treatment with 1-NMPP1 during serum stimulation abolished the phosphorylation of CDK4 bound to cyclin D1, cyclin D3 or p21 in K7AS but not in wt HCT116 cells (Figure 1D). Consistent with our previous observations [33], CDK6 was less phosphorylated than CDK4 and its T177-phosphorylated form was detected in cyclin D1-CDK6 but not in cyclin D3-CDK6. Phosphorylation of cyclin D1-bound CDK6 was also inhibited in response to CDK7 inhibition (Figure S2B).

**Figure 2:**
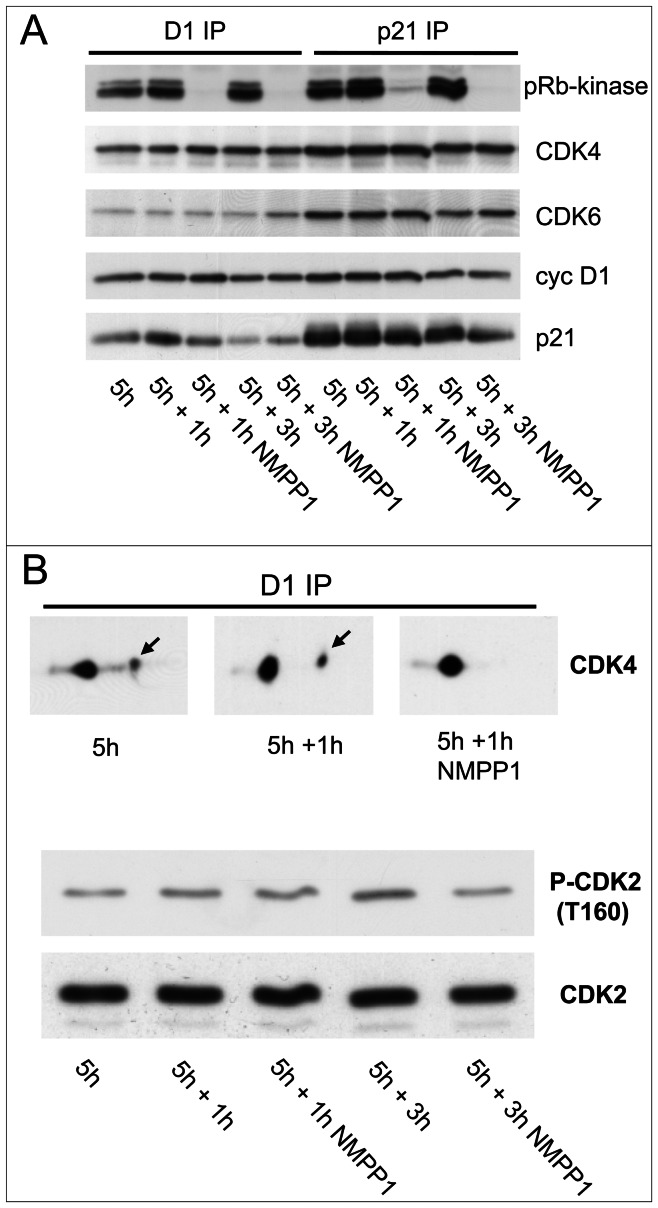
Effect of acute inhibition of CDK7 by 1-NMPP1. Effect of acute inhibition of CDK7 by 1-NMPP1 on CDK4 activity (A) and phosphorylation (B). (A,B) HCT116 K7AS cells were stimulated with FBS for 5 h and 1-NMPP1 was then added for 1 or 3 h. Cell lysates were immunoprecipitated (IP) with anti-cyclin D1 (D1), or anti-p21 antibodies, assayed for their pRb-kinase activity, separated by SDS-PAGE and immunoblotted with the indicated antibodies (A), or separated by 2D gel electrophoresis followed by CDK4 immunodetection (B). In (B), T160 phosphorylation of CDK2 immunodetected from whole cell extracts is shown for comparison.

Application of CDK7 inhibition after mitogenic stimulation has been reported to have only a delayed effect on CDK2 activity because T160 phosphorylation of CDK2 is stabilized by cyclin binding [53]. We thus wanted to compare the stability of the activating phosphorylations of CDK2 and CDK4, and of CDK4 activity, in response to shorter treatments with 1-NMPP1 in K7AS cells. Serum-deprived K7AS cells were stimulated for 5 h before treating them for 1 or 3 h with 1-NMPP1. Both the CDK4/6 pRb kinase activity (Figure 2A) and CDK4 phosphorylation (Figure 2B) completely disappeared after 1 h of CDK7 inhibition. By contrast, T160 phosphorylation of CDK2 was only partially reduced 3 h after 1-NMPP1 administration (Figure 2B).

These results show that the activation of CDK4 and CDK6 *in vivo* depends critically on CDK7 activity. Moreover, CDK4 phosphorylation is labile and thus more prone to rapid regulation, potentially making CDK4 the most influential target of CDK7 in G1/S transition.

### CDK4 complexes from CDK7-inhibited cells are more strongly associated with p21 and are refractory to *in vitro* activation by CAK

In co-immunoprecipitations using antibodies against cyclin D1 and cyclin D3, we noticed a marked increase in the association of p21 in response to sustained CDK7 inhibition. This increase was not paralleled by a similar increase of CDK4 and CDK6 associations (lanes 3 and 5 in Figure 1C). Interestingly, the increased association of p21 was already observed at the 5 h time point, when the total p21 level had not yet increased in response to CDK7 inhibition (Figure 1B, 1C). Because in serum-stimulated HCT116 cells in G1 (which express relatively higher p21 levels than other cells) most of CDK4 and CDK6 complexes are stabilized by p21 and hence contain it, the increased p21 binding in response to CDK7 inhibition indicated an increased stoichiometry (and/or stability) of p21 binding to cyclin D complexes. Binding of more than one p21 molecule is known to preclude the activity of cyclin D-CDK4-p21 complexes [3], [6], [10]. Therefore, we designed experiments to evaluate the extent to which this increased association of p21 could contribute to inhibition of the activation of CDK4/6 caused by CDK7 inhibition.

We first evaluated if CDK4 and CDK6 complexes from CDK7-inhibited cells can be re-activated by CAK. Indeed, if the impaired activation of CDK4 and CDK6 complexes in CDK7-inhibited K7AS cells was due only to the absence of activating phosphorylation, these complexes should remain phosphorylatable by CAK. We and others have shown that cyclin D3-CDK4 can be phosphorylated and activated *in vitro* by cyclin H-CDK7-MAT1 (CAK) complexes [15], [31], [46]. Nevertheless, this is a relatively inefficient process, which requires higher (millimolar) ATP concentrations [31], [33], compared with *in vitro* phosphorylation of CDK2 and cyclin D3-CDK6 [32], [42], [49].

Cyclin D1 and cyclin D3 immunoprecipitates from K7AS cells –which contained mostly unphosphorylated CDK4 and CDK6 after serum-deprivation or re-stimulation by serum in the presence of 1-NMPP1– were incubated with 2 mM ATP and active recombinant CAK. As shown in Figure S3 and detailed in its legend, only cyclin D3-CDK6, but neither cyclin D3-CDK4, cyclin D1-CDK4 nor cyclin D1-CDK6, could be phosphorylated by CAK from 1-NMPP1-treated cells. However, as a positive control in the same experiment, CAK efficiently phosphorylated cyclin D3-CDK4 complexes that were produced in CHO cells (and hence were not associated with p21 [33]) and were dephosphorylated by λ-phosphatase (Figure S3, inset). The refractoriness to CAK activation of cyclin D3-CDK4 from CDK7-inhibited cells (Figure S3) might have been due to its increased association with p21 (Figure 1C). Indeed, p21-bound CDK4 and CDK6 from CDK7-inhibited cells were also refractory to phosphorylation by CAK (Figure S3).

### CDK4 activation correlates with S130 phosphorylation of p21, which is prevented by CDK7 inhibition

**Figure 3:**
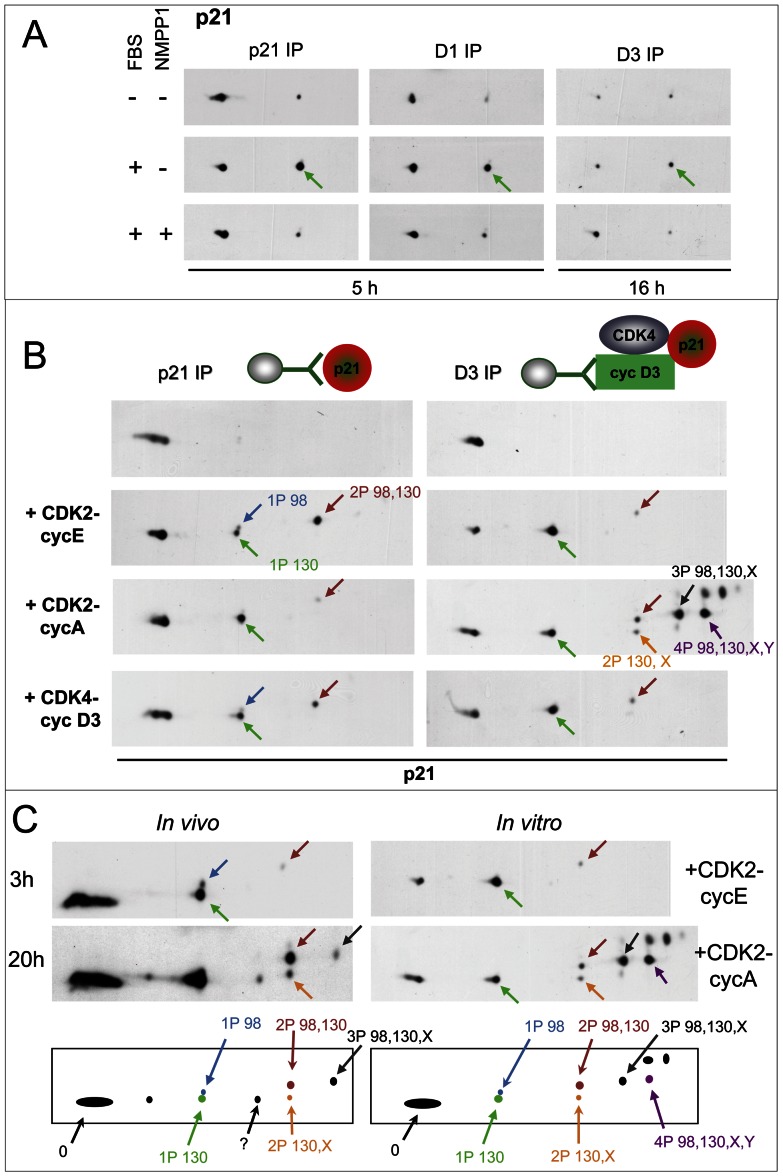
Specific inhibition of CDK7 by 1-NMPP1 prevents the main phosphorylation of p21, which is performed *in vitro* by both CDK4 and CDK2. (A) HCT116 K7AS cells were stimulated (+) or not stimulated (−) with fetal bovine serum (FBS) for the indicated times in the absence (−) or presence (+) of 1-NMPP1. Cell lysates were immunoprecipitated (IP) with anti-p21, anti-cyclin D1 (D1) or anti-cyclin D3 (D3) antibodies and separated by 2D gel electrophoresis followed by p21 immunodetection. (B) CHO cells were transfected with a p21-expressing plasmid alone (left) or with plasmids encoding cyclin D3, CDK4-HA and p21 (right). Cell lysates were immunoprecipitated (IP) with anti-p21 (p21) or anti-cyclin D3 (D3) antibodies and incubated with the indicated recombinant kinases and ATP, before separation by 2D gel electrophoresis and p21 immunodetection. Arrows indicate the main phosphorylated forms of p21 as identified in Figure S4. (C) Schematic representation and comparison of p21 patterns from HCT116 K7AS cells that were restimulated for 3 and 20 h (*in vivo*) and from cyclin D3-CDK4-p21 complexes produced in CHO cells and phosphorylated by recombinant CDK2-cyclin E and CDK2-cyclin A (*in vitro*). The observed p21 forms are labeled according to their number of phosphorylations and the identified phosphorylated residues (e.g., 2P 98,130 means p21 phosphorylated at both S98 and S130; X, Y, unidentified phosphorylated sites).

To better understand how p21 could intervene in the regulation of CDK4 activation even before the increase of p21 steady-state level, we evaluated p21 modifications in K7AS cells by 2D gel electrophoresis. As shown in Figure 3A, one main more negatively charged form appeared in response to serum stimulation in both total p21 and p21 bound to cyclin D1 and cyclin D3 complexes. More modified minor forms in stimulated cells were detected by longer exposure of the western blots (Figure S4A). λ-phosphatase treatment showed that all the modified forms resulted from phosphorylation (not shown). The main single phosphorylated p21 form was induced 2–3 h after serum stimulation, coinciding with the appearance of the T172-phosphorylated form of CDK4 (Figure S4C). During the reduction of p21 levels associated with late G1 and S-phase progression, this phosphorylated form became the most abundant one. Later (16–20 h), coinciding with cyclin A expression (Figure S1B), the minor multi-phosphorylated forms of p21 became more abundant (Figure S4C). Importantly, inhibition of CDK7 by 1-NMPP1 prevented the appearance of the main and minor (multi) phosphorylated forms of p21 (Figure 3A; Figure S4A). As observed for CDK4 phosphorylation (Figure 2B), the phosphorylated forms of p21 already disappeared 1 h after 1-NMPP1 administration (Figure S4B).

We next wanted to identify the phosphorylation sites of p21 in K7AS cells. Different phosphorylated forms of a protein present characteristic reproducible migrations in 2D gel separations, which allows them to be monitored once they are identified [41]. As the phosphorylations of p21 depended on CDK7 activity, we hypothesize that they could have been catalyzed by CDKs, which phosphorylate S/T-P motifs. p21 contains three such motifs (T57, S98, S130). So we produced human p21 and its T57A, S98A and S130A mutants in CHO cells, and compared their 2D gel separation patterns after *in vitro* phosphorylation by active recombinant CDK complexes. In the same experiments, p21 was also produced as a complex with cyclin D3 and CDK4 (immunoprecipitated by the DCS-28 cyclin D3 antibody) to evaluate how the accessibility of p21 phosphorylation sites might have been affected. As analyzed in Figure 3B and Figure S5, cyclin E1-CDK2 and cyclin D3-CDK4 phosphorylated unbound p21 at both S130 and S98 but not at T57 (the patterns were not affected by the T57A mutation), whereas only S130 was phosphorylated by cyclin A2-CDK2. In p21 complexed with cyclin D3 and CDK4, phosphorylation of S130 by cyclin E1-CDK2 and cyclin D3-CDK4 was more complete, while S98 phosphorylation was much reduced. By contrast, after incubation with cyclin A2-CDK2, cyclin D3/CDK4-bound p21 was more completely phosphorylated and several multi-phosphorylated forms appeared. The S130A mutation affected all the phosphorylated forms (Figure S5B). The multi-phosphorylated forms were similarly affected by the S98A mutation, whereas the T57A mutation had no effect (Figure S5B). Therefore, after phosphorylation by cyclin A2-CDK2, all phosphorylated forms of cyclin D3/CDK4-bound p21 contained the S130 phosphorylation, and its most abundant forms resulted from a combination of S130 and S98 phosphorylations associated with one or two unidentified phosphorylations (but not on T57) (Figure 3B; Figure S5). In similar *in vitro* experiments, the association of p21 with cyclin D1-CDK4 was also required for its complete S130 phosphorylation by cyclin D1-CDK4 and cyclin D1-CDK6 (Figure S6). Overall, these observations suggest that interaction of p21 with cyclin D-CDK4 increases the accessibility of the S130 residue to the five recombinant active CDKs we tested, and the accessibility of S98 to cyclin A2-CDK2 only, while impairing S98 accessibility to cyclin E1-CDK2 and cyclin D1/D3-CDK4. CDK4 was not phosphorylated by recombinant CDK2 and CDK4 complexes in these experiments (not shown).

Comparison of *in vitro* and *in vivo* phosphorylation patterns of p21 (Figure 3C) showed identical migration of forms generated by cyclin E1-CDK2 and cyclin D1/D3-CDK4/6 with those that appeared in K7AS cells at time points corresponding to G1 and early S-phase progression (see also Figure S4C). This indicates that p21 was mostly phosphorylated at S130 and S98 (the single S130-phosphorylated form being the most abundant one). Additional multi-phosphorylated p21 forms that appeared during late S and the G2 phase comigrated identically with those generated by phosphorylation of cyclin D3/CDK4-bound p21 by cyclin A2-CDK2 (Figure 3C). As also confirmed by p21 mutation in intact cells (see below), the phosphorylation of p21 induced by mitogenic stimulation and inhibited by CDK7 inhibition occurred mainly at residue S130.

**Figure 4:**
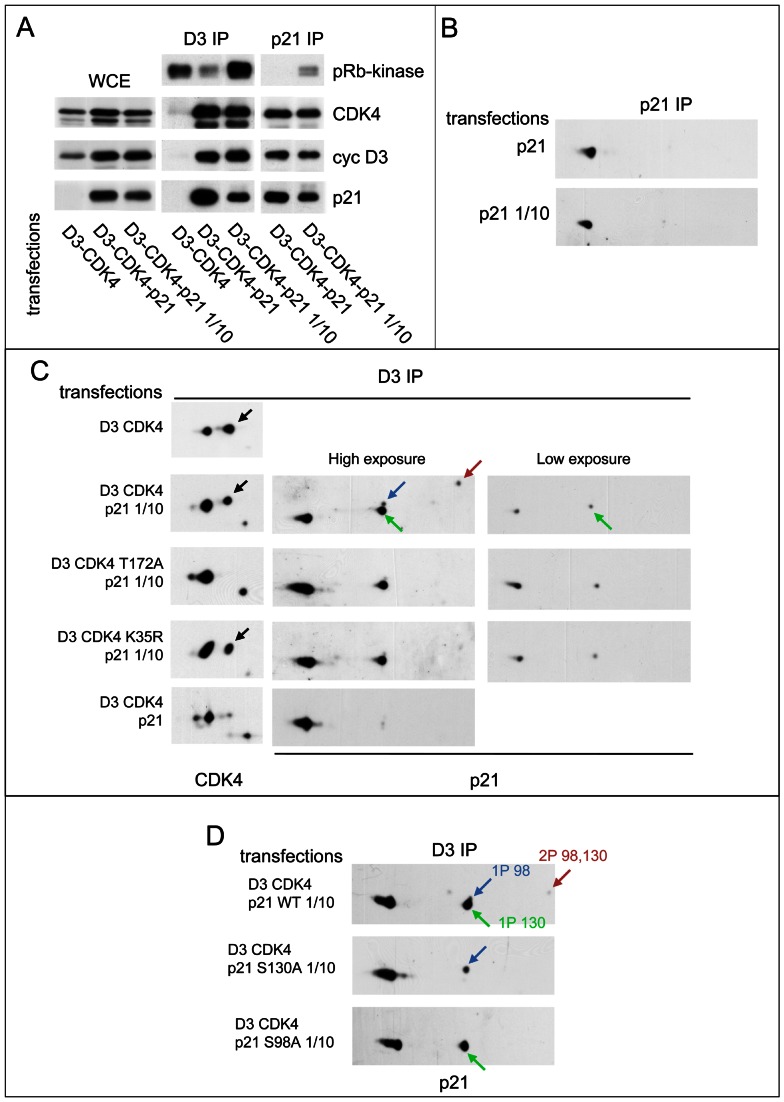
Reciprocal impacts of p21 and cyclin D3-CDK4 on T172 phosphorylation of CDK4 and S130 phosphorylation of p21 in transfected CHO cells. (A) p21 stabilizes cyclin D3-CDK4 complexes and inhibits their activity depending on its expression level. CHO cells were transfected with plasmids encoding cyclin D3 and CDK4-HA with or without p21 at high (p21) or low (p21 1/10) expression level. Cell lysates (WCE) were immunoprecipitated (IP) with anti-cyclin D3 (D3) or anti-p21 antibodies, assayed for their pRb-kinase activity, separated by SDS-PAGE and immunoblotted with the indicated antibodies. (B) p21 expressed alone is essentially unphosphorylated. CHO cells were transfected with a plasmid encoding p21 at high (p21) or low (p21 1/10) expression level. Cell lysates were immunoprecipitated (IP) with anti-p21 antibody and separated by 2D gel electrophoresis followed by p21 immunodetection. (C) Co-expression of cyclin D3-CDK4 induces the phosphorylation of p21, largely independently of CDK4 activity, whereas stronger expression of p21 inhibits phosphorylations of both p21 and CDK4. CHO cells were transfected with plasmids encoding cyclin D3 and CDK4-HA or its inactive mutants (T172A, K35R) with or without p21 at high (p21) or low (p21 1/10) expression level. Cell lysates were immunoprecipitated (IP) with anti-cyclin D3 antibody and separated by 2D gel electrophoresis followed by CDK4 and p21 immunodetection. (D) Identification of p21 phosphorylation sites. CHO cells were transfected with plasmids encoding cyclin D3, CDK4-HA and wild type (WT) or mutated (S130A, S98A) p21 at low (p21 1/10) expression level. Cell lysates were immunoprecipitated (IP) with anti-cyclin D3 antibody and separated by 2D gel electrophoresis followed by p21 immunodetection. Black arrows, T172-phosphorylated CDK4. Colored arrows indicate phosphorylated forms of p21.

To further evaluate the relationship between p21 and CDK4 T172 phosphorylation in intact cells, p21 was co-expressed at two different levels with cyclin D3 and CDK4 by transfection of CHO cells (in which no endogenous p21 is detectable) (Figure 4). The effect of p21 expression on the assembly and activity of CDK4 complexes was exactly as established [10], [58]–[60]. In the absence of p21, very active cyclin D3-CDK4 complexes were formed [31], and they were largely phosphorylated at T172 (Figure 4A, 4C). At the higher level of expression, p21 stabilized cyclin D3-CDK4 complexes but inhibited their pRb-kinase activity (Figure 4A) and also substantially reduced CDK4 phosphorylation (Figure 4C). By contrast, at the lower expression level of p21, p21-bound cyclin D3-CDK4 complexes displayed a weak but readily detected activity (Figure 4B) and a much increased phosphorylation of CDK4 (Figure 4C).

When expressed alone, whether at a high or a low level, p21 was essentially unphosphorylated (Figure 4B). Intriguingly, in the “low p21” setting, the co-expression of cyclin D3-CDK4 induced the phosphorylation of p21 at S130 and less abundantly at S98 (as shown by comparison with S130A and S98A mutants) (Figure 4C, 4D). This effect of cyclin D3-CDK4 on S130 phosphorylation mostly did not depend on the activity of the CDK4 complex, as observed by comparison with two inactive CDK4 mutants, T172A and K35R (Figure 4C). On the other hand, in the “high p21” setting, p21 and CDK4 were essentially unphosphorylated (Figure 4C), and the relative presence of p21 was also increased in the CDK4 complex (Figure 4A, compared to the “low p21” setting in the cyclin D3 immunoprecipitation).

All together, experiments on transfected CHO cells further established the correlation between T172 phosphorylation of CDK4 and S130 phosphorylation of p21. Binding of p21 to cyclin D3-CDK4 might expose S130 to another kinase, as also suggested by the previous *in vitro* experiments. That this p21 S130-kinase could itself be inhibited by p21 is suggested by the observation that S130 phosphorylation of p21 was not induced in cyclin D3-CDK4 complexes when p21 was expressed at concentrations that largely exceed the binding capacity of the CDK4 complex.

Previous reports have indicated that S130 phosphorylation of p21 can be catalyzed by CDK2 to form a phosphodegron recognized by the SCF/Skp2 ubiquitin ligase complex and to signal the proteasomal degradation of cyclin/CDK-bound p21, which is required for S-phase entry [61]–[63]. To evaluate whether inhibition of proteasomal degradation of p21 could indeed affect CDK4 phosphorylation, K7AS HCT116 cells were stimulated by serum in the presence or absence of the proteasome inhibitor MG132 (Figure S7). MG132 increased p21 levels (Figure S7A) and strongly inhibited the pRb-kinase activity associated with cyclin D1 and p21 (Figure S7B). Here also, both the T172 phosphorylation of CDK4 and the S130 phosphorylation of p21 were inhibited by MG132 (Figure S7C).

### S130 phosphorylation of p21 is instrumental in CDK4 activation

**Figure 5:**
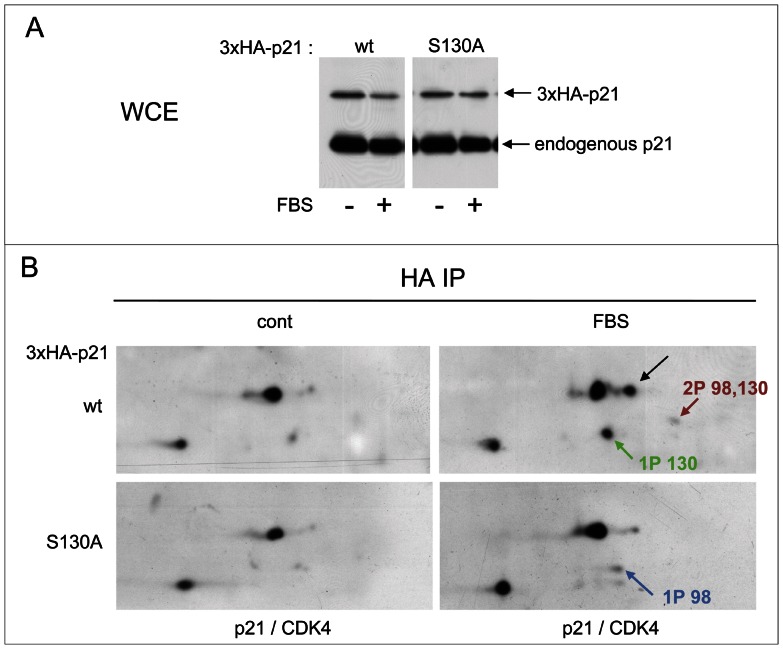
S130 phosphorylation of p21 is instrumental in CDK4 activation. (A,B) Stably infected HCT116 K7AS cells for Tet-On inducible 3×HA-p21 wt or S130A mutant were treated with doxycycline (1 µg/ml) for 16 h prior cell restimulation with fetal bovine serum (FBS) for 16 h in the continuous presence of doxycycline. (A) Immunodetection of 3×HA-p21 and endogenous p21 from whole cell extracts (WCE) using a p21 antibody. (B) Cell lysates were immunoprecipitated (IP) with anti-HA antibody and separated by 2D gel electrophoresis followed by simultaneous immunodetection of ectopic 3×HA-p21 and 3×HA-p21-bound endogenous CDK4 using a mixture of anti-CDK4 and p21 antibodies. Black arrows, T172-phosphorylated CDK4. Colored arrows indicate phosphorylated forms of p21.

To evaluate the effect of the S130 phosphorylation of p21 on the activation of CDK4 in HCT116 cells, we generated by lentiviral infection TetON-inducible K7AS cells that inducibly express 3×HA-tagged wt or S130A p21. 3×HA-p21 was induced by doxycycline and the cells were stimulated by serum. Upon induction by doxycycline, ectopic p21 expression from puromycine-selected cells was maintained well below the level of endogenous p21 and it was identical in wt and S130A 3×HA-p21 cells (Figure 5A). From these cells, ectopic p21 was immunoprecipitated with an HA antibody and the immunoprecipitates were separated by 2D-gel electrophoresis to analyze the phosphorylations of 3×HA-p21 and CDK4 bound to the ectopic p21 (co-detected using a mixture of p21 and CDK4 antibodies). As shown in Figure 5B, in serum-stimulated cells the main singly phosphorylated form of p21 was abrogated by the S130A mutation, confirming its identification. Moreover, CDK4 phosphorylation was much reduced by the S130A p21 mutation (Figure 5B). Therefore, inhibition of S130 phosphorylation of p21 could really contribute to impairment of CDK4 activation.

Additional experiments were performed to understand how S130 phosphorylation could affect the activation of p21-bound cyclin D-CDK4 complexes (detailed in Figure S8 and its legend). Since the increased association of p21 to CDK4 complexes in response to CDK7 inhibition occurred before the increased accumulation of overall p21 (Figure 1B, 1C), we envisaged an effect of the S130 phosphorylation on the stability of the interaction of p21 with CDK4 complexes. p21 interacts with cyclin-CDK complexes through two cyclin-binding sites, designated Cy1 and Cy2, respectively located in the N and the C terminus, and a single CDK-binding site (K) closer to the central region [64]. Inducible expression of the S130D “phosphomimetic” 3×HA-p21 mutant in K7AS HCT116 cells did not reproducibly affect the binding of p21 to CDK4 complexes or CDK4 phosphorylation (Figure S8A). However, in the presence of another p21 mutation that affects the K CDK-binding site (T57D; [65] and our unpublished data), the S130D mutation markedly reduced the co-immunoprecipitation of CDK4 with 3×HA-p21 (Figure S8A). Interestingly, CDK4 complexed with the double T57D/S130D p21 mutant was also more abundantly phosphorylated than CDK4 bound to T57D p21 (Figure S8A). Finally, as shown in Figure S8B and detailed in its legend, we also tested the effect of *in vitro* phosphorylation of p21 by CDK2 on the subsequent phosphorylation of the p21-bound CDK4 by CAK. In the T57D mutation context, *in vitro* phosphorylation by cyclin A2-CDK2 reduced p21 interaction with CDK4, thereby allowing complete phosphorylation by CAK of the remaining p21-bound CDK4 (Figure S8B).

All together, these experiments demonstrate that S130 phosphorylation of p21 is required for phosphorylation of p21-bound CDK4. Moreover, they suggest that S130 phosphorylation might modify the interaction of p21 with cyclin D-CDK4 to increase the accessibility of CDK4 T172 to CAK (and possibly to other kinases).

### CDK2 and CDK4/6 activities are both required for S130 phosphorylation of p21 and CDK4 activation

**Figure 6:**
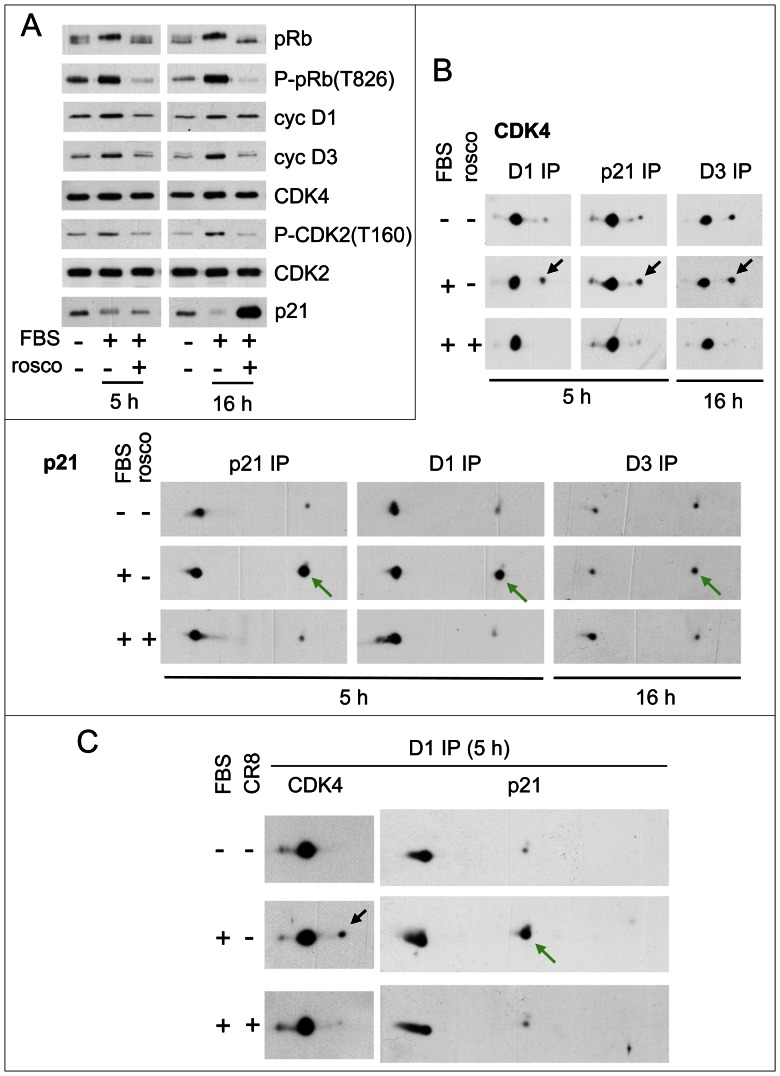
Inhibition of phosphorylations of p21 and CDK4 by CDK inhibitors. HCT116 K7AS cells were stimulated (+) or not stimulated (−) with fetal bovine serum (FBS) for the indicated times in the absence (−) or presence (+) of roscovitine (rosco) (A,B) or CR8 (C). (A) Western blotting analysis with the indicated antibodies from whole-cell lysates. (B,C) Cell lysates were immunoprecipitated (IP) with anti-cyclin D1 (D1), anti-cyclin D3 (D3) or anti-p21 antibodies and separated by 2D gel electrophoresis followed by immunodetection of CDK4 and p21. Black arrows, T172-phosphorylated CDK4; green arrows, S130-phosphorylated p21.

*In vitro*, p21 bound to cyclin D-CDK4 was efficiently phosphorylated at S130 by cyclin E- CDK2 and cyclin A-CDK2 complexes, and in transfected CHO cells S130 was phosphorylated by p21-inhibitable kinase(s). To ascertain the possible involvement of CDK2 in S130 phosphorylation of p21 and hence in CDK4 activation, we first stimulated K7AS HCT116 cells in the presence or absence of the specific CDK inhibitor roscovitine, which *in vitro* inhibits several CDKs but not CDK4 and CDK6 [66]. Using a concentration of R-roscovitine (20 µM) that has little effect on cell cycle and survival in CDK2-null MEFs [66], we observed a strong inhibition of pRb phosphorylation, including at the T826 CDK4/6-specific site. The effect was already observed in G1 phase (5 h after stimulation), and at that time it was not associated with any major changes in the expression of p21 (Figure 6A; noteworthy, p21 was strongly induced by roscovitine at later time points). Here again, the T172 phosphorylation of CDK4 as well as S130 phosphorylation of p21 were inhibited by roscovitine (Figure 6B). Nevertheless, T160 phosphorylation of CDK2 was also reduced (Figure 6A), suggesting that roscovitine might have inhibited CDK7 in addition to CDK2, as reported previously [67].

Therefore, we also used S-CR8 (0.5 µM), a more potent and selective derivative of roscovitine, with a 20-fold lower *in vitro* IC_50_ for inhibition of CDK2 than CDK7 [68]. Analysis of the phosphorylations of the C-terminal domain (CTD) of the large subunit of RNA polymerase II indeed confirmed that CR8 likely inhibited CDK9 in addition to CDK2, but unlike roscovitine, not CDK7 (Figure S9A and its legend). In the same experiments, CR8 inhibited the serum-stimulated phosphorylation of p21 and CDK4 (Figure 6C), further supporting the involvement of CDK2.

We then compared the short term effect on CDK4 activation of roscovitine, CDK7 inhibition by 1-NMPP1, and CDK2 inhibition by CR8. Whereas continuous treatment with CR8 abolished CDK4 activation (Figure 6C), CDK7 inhibition and roscovitine inhibited CDK4 activity (not shown) and phosphorylation in 1 h, but CR8 did not (Figure S9B). Therefore, the effect of CDK2 inhibition was delayed compared to the abrupt arrest of CDK4 activation resulting from CDK7 inhibition, suggesting that CDK7 inhibition can affect CDK4 activation by both indirect (CDK2-mediated) and more rapid (CDK2-independent) mechanisms. This result also confirmed that CR8 did not affect CDK4 activation by inhibiting CDK7.

Finally, we wanted to exclude the possibility that CDK2 inhibition by CR8 can indirectly affect CDK7 activity. Based on *in vitro* experiments, CDK2 was indeed proposed to be responsible for T170 phosphorylation of CDK7 [51]. As shown in Figure S10A, the capacity of cyclin H-CDK7 complexes of HCT116 K7AS cells to activate cyclin D3-CDK4 *in vitro* was affected only slightly by stimulating these cells with serum (confirming previous reports from other cell systems [36], [37], [46]) or inhibiting CDK2 by CR8. In K7AS cells, the association of CDK7 and XPD [69] with cyclin H was not modified by these treatments, although MAT1 association was slightly increased by serum stimulation (Figure S10A). Furthermore, by combining 2D electrophoresis and a phosphospecific T170 CDK7 antibody, we found that almost all cyclin H-bound CDK7 was phosphorylated at T170 as well as at a second site, likely S164 [51]. This CDK7 phosphorylation profile was not affected in cells continuously treated with CR8 (Figure S10B). Therefore, the proposed mechanisms of CDK7 regulation [50] cannot explain the regulation of CDK4 T-loop phosphorylation and the effect of CR8 in our experiments.

**Figure 7:**
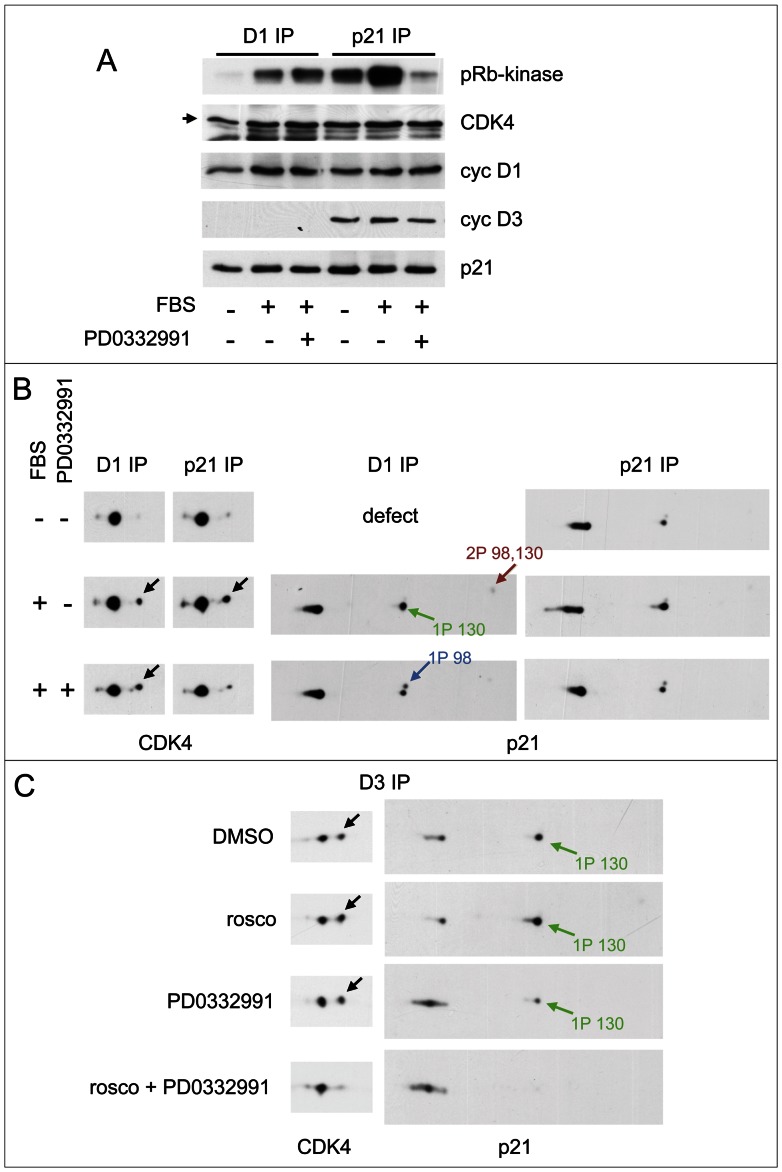
Activities of both CDK4/6 and CDK2 are required for S130 phosphorylation of p21 and activation of p21-bound CDK4. (A,B) HCT116 K7AS cells were stimulated (+) or not stimulated (−) with fetal bovine serum (FBS) for 5 h in the absence (−) or presence (+) of the CDK4/6 inhibitor PD0332991. Cell lysates were immunoprecipitated (IP) with anti-cyclin D1 (D1) or anti-p21 antibodies, assayed for their pRb-kinase activity, separated by SDS-PAGE and immunoblotted with the indicated antibodies (A), or separated by 2D gel electrophoresis followed by CDK4 and p21 immunodetection (B). (C) CHO cells were transfected with plasmids encoding cyclin D3, CDK4-HA and p21 at low (p21 1/10) expression level (see Figure 4), in the absence (DMSO as vehicle) or presence of CDK inhibitors: roscovitine (rosco), PD0332991 (1 µM) or their combination to inhibit both CDK2 and CDK4. Cell lysates were immunoprecipitated (IP) with anti-cyclin D3 (D3) antibody and separated by 2D gel electrophoresis followed by CDK4 and p21 immunodetection. Black arrows, T172-phosphorylated CDK4; colored arrows, phosphorylated forms of p21.

We observed that CDK4 and CDK6, associated with cyclin D3 and cyclin D1, also phosphorylated p21 complexed with D-type cyclins and CDK4 *in vitro* (Figure 3B; Figure S6). So we evaluated the effect of PD0332991 on S130 phosphorylation and CDK4 activation in K7AS HCT116 cells (Figure 7). PD0332991 is a specific inhibitor of CDK4 and CDK6 and does not inhibit CDK2 and the proliferation of pRb-negative cells [27], [70]. PD0332991 (250 nM) inhibited the T826 phosphorylation of pRb (not shown) and partially the S130 phosphorylation of p21, but apparently not the S98 phosphorylation (Figure 7B). Treatment of the cells with PD0332991 did inhibit the phosphorylation and consequently *in vitro* activity of p21-bound CDK4 (Figure 7A, 7B). This indicates that this drug inhibited not only the activity of CDK4 but also its activation by phosphorylation, which might be related to the inhibition of p21 phosphorylation. Curiously, and at variance with all the inhibitory treatments we used, such inhibitory effects of PD0332991 cell treatment on CDK4 activation were not observed for CDK4 precipitated by antibodies against cyclin D1 (Figure 7A, 7B) and cyclin D3 (not shown). This puzzling and very reproducible discrepancy necessarily implies that in PD0332991-treated cells the active phosphorylated cyclin D1/3-bound CDK4 was no longer associated with p21. We speculate that high-affinity binding of this ATP-competing drug to CDK4 in cells had somehow impaired p21 binding to CDK4, e.g. by competing with the insertion of the 3_10_ helix of p21 in the catalytic cleft of CDK4 or by altering the folding of CDK4. Regardless of the reason, these observations show that the effect of PD0332991 on CDK4 activation depended on the binding of CDK4 to p21. They thus suggest that CDK4 (and/or CDK6) might contribute to the activation of p21-bound CDK4 complexes by phosphorylating p21.

In asynchronously proliferating CHO cells transfected to overexpress cyclin D3 and CDK4 in the presence of a limiting amount of ectopic p21 (performed as in Figure 4), roscovitine treatment was insufficient to reduce the phosphorylations of CDK4 and p21. This intriguing observation is at variance with the situation observed in re-stimulated K7AS cells. So we re-investigated the effect of roscovitine in the presence of CDK4/6 inhibition by PD0332991 in such transfected CHO cells (Figure 7C). Whereas PD0332991 partly inhibited p21 phosphorylation, roscovitine alone was inactive. Only the combination of both inhibitors abrogated both p21 and CDK4 phosphorylations (Figure 7C), suggesting that they depended on both CDK4/6 and CDK2 (and/or CDK7) activities in this experimental setting.

It has been reported that to phosphorylate p21 on S130, cyclin E-CDK2 has to interact with the C-terminus Cy2 cyclin-binding motif of p21 [61]. To evaluate the importance of this motif *in vivo*, we again used the lentiviral Tet-ON inducible system to express a 3×HA-tagged deletion mutant of p21 lacking the Cy2 motif (aa 1 to 154; ΔCy2) in HCT116 cells. As performed in the previous Figure 5 experiment, this mutant and wt p21 were induced by doxycycline and the cells were stimulated by serum. Ectopic p21 was immunoprecipitated with an HA antibody and the immunoprecipitates were separated by 2D-gel electrophoresis to analyze the phosphorylations of 3×HA-p21 and CDK4 bound to the ectopic p21 (co-detected using a mixture of p21 and CDK4 antibodies). As shown in Figure S11, the ΔCy2 p21 mutant was much less phosphorylated in response to serum stimulation than wt p21. Moreover, serum did not increase the phosphorylation of CDK4 bound to the ΔCy2 p21 mutant (Figure S11), as observed with the S130A p21 mutant (Figure 5). Together with the previous report [61], this result suggests that activation of p21-bound CDK4 *in vivo* requires S130 phosphorylation of p21 by cyclin-CDK complexes, which depends on their binding to the Cy2 site of p21.

### The requirement for CDK7 activity is dependent on CDK4 binding to p21, demonstrating the existence of CDK4-activating kinase(s) other than CDK7 in HCT116 cells

**Figure 8:**
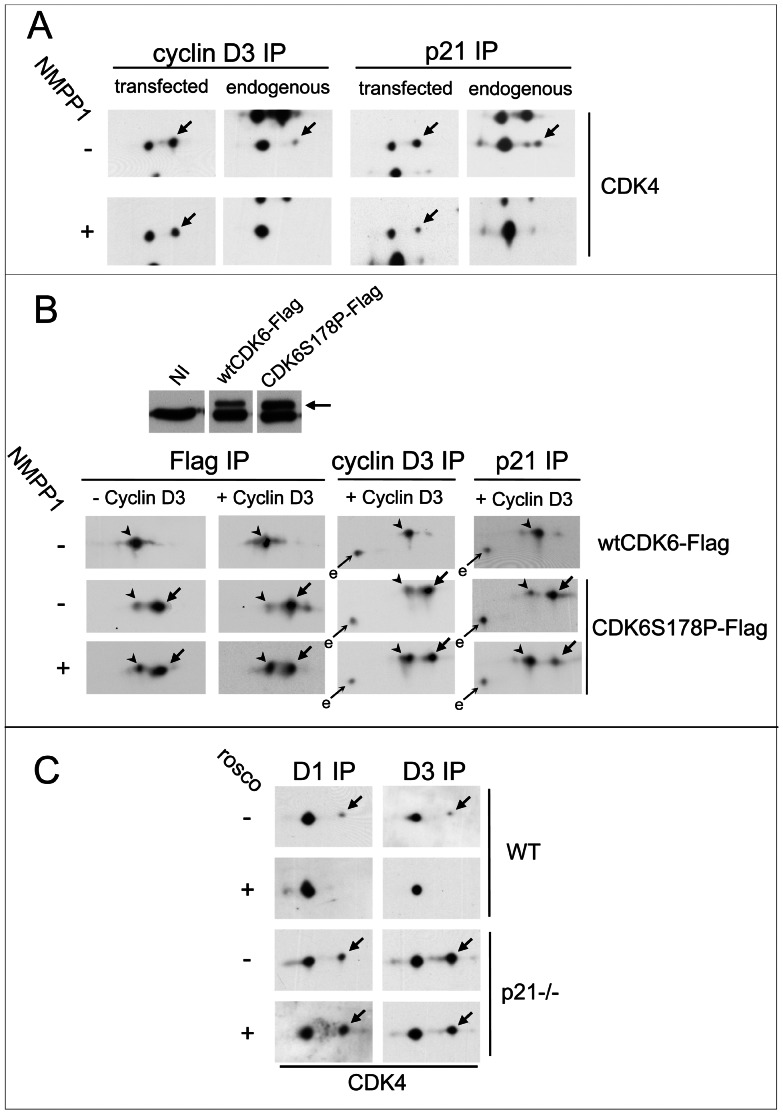
CDK7 inhibition does not prevent activating phosphorylations of p21-unbound CDK4 and S178P CDK6. (A) HCT116 K7AS cells were transfected with plasmids encoding cyclin D3 and CDK4-HA in the absence or continuous presence of 1-NMPP1 for 48 h. Cell lysates were immunoprecipitated (IP) with anti-cyclin D3 or anti-p21 antibodies and separated by 2D gel electrophoresis followed by immunoblotting with anti-CDK4 antibody, which detected both ectopic and endogenous CDK4. (B) HCT116 K7AS cells were infected during 24 h by lentiviruses encoding Tet-On inducible wt or S178P CDK6-Flag with or without lentiviruses encoding Tet-On inducible cyclin D3. The expression of these proteins in the continuous presence (+) or absence (−) of 1-NMPP1 was induced by doxycycline 16 h prior to cell restimulation by fetal bovine serum (FBS) for 16 h. Western blotting detection of endogenous and ectopic (arrow) CDK6 from whole cell extracts is shown in the upper panel (NI, not infected). Cell lysates were immunoprecipitated (IP) with anti-Flag, anti-cyclin D3 or anti-p21 antibodies and separated by 2D gel electrophoresis followed by immunoblotting with anti-CDK6 antibody, which detected both ectopic and endogenous (e) CDK6. (C) T172 phosphorylation of endogenous CDK4 resists inhibition of CDK2 and CDK7 by roscovitine in p21-null (p21−/−) cells. Roscovitine (rosco; +) or vehicle (−) were administered for 16 h to asynchronously growing wild-type (WT) and p21−/− HCT116 cells cultured in parallel. Cell lysates were immunoprecipitated (IP) with anti-cyclin D1 (D1) or anti-cyclin D3 (D3) antibodies and separated by 2D gel electrophoresis followed by CDK4 immunodetection. Arrows, phosphorylated forms of CDK4 and CDK6-Flag; arrowheads in (B), unphosphorylated form of CDK6-Flag.

We next wanted to verify the effect of CDK7 inhibition under the experimental conditions that permitted us to propose the existence of non-CDK7 proline-directed CDK4-activating kinases, i.e. using ectopic cyclin D3-CDK4 overexpressed by cell transfection as previously [33]. HCT116 K7AS cells were co-transfected using expression plasmids encoding cyclin D3 and HA-tagged CDK4 in the continuous presence or absence of 1-NMPP1. The HA-tag allowed us to distinguish endogenous and ectopic CDK4 after 2D gel separation from the same co-immunoprecipitations. As shown in Figure 8A, cyclin D3-bound CDK4-HA was more phosphorylated than endogenous CDK4, and unlike endogenous CDK4, its phosphorylation was largely resistant to CDK7 inhibition. However, in the minor fraction of CDK4-HA that could be co-immunoprecipitated through its binding to endogenous p21 (using a p21 antibody), we observed a partial but marked reduction of CDK4 phosphorylation after CDK7 inhibition by 1-NMPP1 (Figure 8A). Therefore, we reasoned that the higher T172 phosphorylation level and its resistance to CDK7 inhibition seen in most ectopic cyclin D3-CDK4 complexes might have been due to their overexpression, which allows them to assemble without being stabilized by the endogenous p21, which was not present in sufficient amounts.

We evaluated this possibility in a setting that did not depend on CDK overexpression. We have previously shown that the “CDK4-mimicking” S178P mutation of CDK6 dramatically increases its activity by inducing its almost complete T177 phosphorylation. Interestingly, phosphorylation of S178P CDK6 does not require cyclin binding (at variance with CDK4) [33], and so it is not influenced by binding to a Cip/Kip protein, which requires prior association with a cyclin [71]. By lentiviral infection, we generated TetON-inducible K7AS cells that express Flag-tagged wt or S178P CDK6, with or without cyclin D3, in response to doxycycline (Figure 8B). In sharp contrast with wt Flag-CDK6, the induced Flag-S178P CDK6 was almost completely T177-phosphorylated, as shown by its previously characterized [33] 2D gel separation (Figure 8B). The continuous presence of up to 30 µM of 1-NMPP1 to completely inhibit CDK7 activity during the induction by doxycycline only minimally affected the phosphorylation of S178P CDK6 expressed alone (without cyclin D3) (Figure 8B). By contrast, when S178P CDK6 was co-induced with cyclin D3 to allow its interaction with endogenous p21, inhibition of CDK6 phosphorylation was much more pronounced in the fraction of S178P CDK6 co-immunoprecipitated by the p21 antibody from CDK7-inhibited cells (Figure 8B), exactly as observed for p21-bound ectopic CDK4-HA (Figure 8A).

To definitively establish the involvement of p21-binding in the requirement for CDK7 activity in the activation of endogenous CDK4, we compared the effect of the inhibition of both CDK2 and CDK7 by roscovitine in p21-null HCT116 cells and wt HCT116 cells. As p21-null HCT116 cells did not survive serum deprivation, both wt and p21-null cells were treated for 16 h with roscovitine when cycling asynchronously (Figure 8C). Phosphorylation of CDK4 bound to cyclin D1 or cyclin D3 was much more abundant in p21-null cells (Figure 8C) (as observed for overexpressed cyclin D3-CDK4 in Figure 8A). Importantly, T172 phosphorylation of CDK4 was completely inhibited by roscovitine in wt cells but not in p21-null cells (Figure 8C).

Collectively, our results indicate that in HCT116 cells the crucial requirement for CDK7 activity in activating CDK4 by phosphorylation is dependent on its binding to p21. On the other hand, kinases other than CDK7 should exist to phosphorylate p21-unbound CDK4 and S178P CDK6 during continuous CDK7 inhibition.

## Discussion

**Figure 9:**
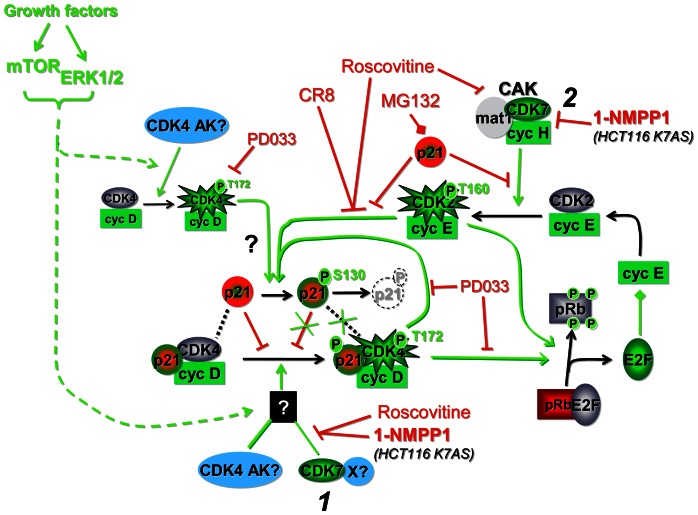
Model assembling the present observations with previous knowledge [6], [10], [37], [53], [61], [62]. CDK7 inhibition reveals novel positive feedbacks mediated by S130 phosphorylation of p21, which is catalyzed by active CDK4 and CDK2 to permit T172 phosphorylation and activation of CDK4 complexes stabilized by p21 binding. S130 phosphorylation of p21 thus amplifies CDK4 activation and subsequently leads to increased degradation of p21, which in turn facilitates CDK2 activation. Those retrocontrols of CDK4 activation should contribute to explaining the irreversibility of the cell cycle commitment at the R point. Green/red colors indicate a final positive/negative influence on R point passage. *1*, *2*, two different levels of CDK7 action demonstrated by the present observations. CDK4 AK?, hypothetical CDK4-activating kinase(s) phosphorylating p21-unbound CDK4 and S178P CDK6 during CDK7 inhibition.

In this study, specific inhibition of CDK7 in the human HCT116 cell line led to several novel observations that collectively reveal that the pRb-E2F switch at the R point is controlled by a complex CDK7-dependent module involving bidirectional functional interactions between CDK4 and CDK2. This process targets the central T172 phosphorylation of CDK4 and is mediated at least partly by S130 phosphorylation of p21. The model depicted in Figure 9 assembles our main observations:

- CDK7 is required for CDK4 activation *in vivo*, but this requirement is conditioned by CDK4 binding to p21, and other CDK4-activating kinases must exist to phosphorylate p21-unbound CDK4 during CDK7 inhibition.
- The action of CDK7 on CDK4 activation is two-pronged. In addition to having an initial acute effect on T172 phosphorylation of CDK4, CDK7 inhibition induced an increased association of p21 to cyclin D-CDK4 complexes, which might have been caused by reduction of the main phosphorylation of p21 at S130. As shown by the impairment of CDK4 phosphorylation by the S130A mutation of p21, the inhibition of S130 phosphorylation likely explains in part the inhibition of the activation of p21-bound CDK4 complexes and the refractoriness of cyclin D3-CDK4 complexes from CDK7-inhibited cells to *in vitro* activation by CAK/CDK7. As reported by others [61], [62], the inhibition of S130 phosphorylation should also explain the subsequent augmentation of overall p21 levels.
- *In vitro*, S130 phosphorylation of p21 was performed not only by cyclin E/A-CDK2 complexes, but also by CDK4 and CDK6 associated with cyclin D1 and cyclin D3. *In vivo*, both S130 phosphorylation of p21 and T172 phosphorylation of p21-bound CDK4 were inhibited not only in response to CDK7 inhibition, but also in response to increased p21 expression and inhibition of CDK2 (by roscovitine and CR8) and CDK4/6 (by PD0332991). Inhibition of CDK4 phosphorylation by roscovitine and PD0332991 depended on p21.

### CDK7 involvement in CDK4 activation

Together with a parallel study by Fisher lab [72], our data extend to CDK4 and CDK6 the concept that CDK7 activity is critically required for the activating phosphorylation of all the CDKs that orchestrate cell cycle progression [53], [73], [74]. As also found in the parallel study [72], CDK4 complexes are much more rapidly deactivated than CDK2 complexes in response to CDK7 inhibition, showing that the T172 phosphorylation of CDK4 is exceptionally labile and thus potentially regulated by phosphatases. Indeed, for CDK4, unlike CDK2, cyclin binding does not block the accessibility of the phosphorylated T-loop to phosphatases [33], [75]. Because of this unique responsiveness of T172 phosphorylation and its initializing role at R point, CDK4 might well be the physiologically most significant target of CDK7 in G1/S transition. On the other hand, in HCT116 K7AS cells and other human cell lines including T98G cells, T-loop (T170) phosphorylation of CDK7 and its activity on CDK4 [35]–[37] were essentially constitutive (Figure S10 and unpublished results). Perhaps due to slight differences in the used cells and/or methodology, we were thus unable to confirm an important stimulation by serum of CDK7 T170 phosphorylation [72].

How could the rapid responsiveness of CDK4 activation to CDK7 inhibition be reconciled with the observations that raised doubts about CAK being the sole CDK4 activating kinase (as summarized in the Introduction; [35])? First, the unaffected phosphorylation of ectopic cyclin D3-CDK4 and S178P CDK6 during CDK7 inhibition, as well as the unaltered phosphorylation of endogenous p21-free CDK4 complexes, demonstrate that CDK7 cannot be the sole CDK4-activating kinase as previously postulated [33], [35]. While completing our study, it was reported that inactivation of Rb family proteins by a SV40 large T fragment restores T-loop phosphorylation of CDK2 and CDK1 despite genetic inactivation of CDK7 [74]. That observation too shows the existence of non-CDK7 CAKs in mammalian cells. Second, in our experiments, the requirement for CDK7 activity appears to be restricted to p21-bound CDK4 (or S178P CDK6). One possible explanation is that p21 binding might be required to co-localize CDK4 in the nucleus with CDK7, the CAK activity of which depends on its nuclear targeting [76]. Outside the nucleus, ectopic cyclin D3-CDK4 and S178P CDK6 (which are mostly cytoplasmic [33]) might be phosphorylated by other proline-directed kinase(s). Third, the requirement for CDK7 activity is complex and at least in part indirect. For example, it might be mediated by CDK2 and CDK4 activation and thus by S130 phosphorylation of cyclin D/CDK4-bound p21, as discussed below. Though the rapid effect of CDK7 inhibition on CDK4 phosphorylation indicates a direct involvement, it does not formally demonstrate it because CDK7 might conceivably act on other CDK4 kinases (or phosphatases). Fourth, CDK7 could still be the catalytic subunit of the regulated proline-directed CDK4-activating kinase that we postulated [33]. *In vitro*, cyclin H-CDK7-Mat1 phosphorylates CDK4 independently of its +1 proline [33], and when coimmunoprecipitated from various cell lysates, it is constitutively active (even when assayed on CDK4) [35]–[37] (Figure S10). Nevertheless, CDK7 might act differently in cells due to interaction with unknown accessory proteins that might not be preserved in CDK7 immunoprecipitations. Such regulated accessory proteins might mediate the complex regulations of CDK4 phosphorylation, including its differential regulation in cyclin D1 and cyclin D3 complexes [36], [47]. Even the possibility that CDK7 would not utilize cyclin H to activate CDK4 in cells cannot be excluded.

### Involvement of S130 phosphorylation of p21 in CDK4 activation and cell cycle progression

The S130 phosphorylation site of p21 is not conserved in p27 and p57. Nevertheless, S130 phosphorylation of p21 has been proposed [62] as the functional homologue of T187 phosphorylation of p27 [12], [77] and T310 phosphorylation of p57 [78]. S130 phosphorylation of p21 has been reported to be similarly catalyzed by CDK2 to form a phosphodegron recognized by the SCF/Skp2 ubiquitin ligase complex and to signal the proteasomal degradation of cyclin/CDK-bound p21 required for S-phase entry [61]–[63]. It was also found to be essential for release of a p21-imposed G1 arrest [79]. Here we show for the first time that this phosphorylation is abundant in total and CDK4-bound p21 during G1 phase, indicating that it does not rapidly lead to p21 degradation at this stage, possibly because Skp2 levels are kept low in G1 by proteasomal degradation initiated by APC^Cdh1^ [63]. Whereas most active cyclin D-CDK4 complexes are associated with p21 in G1-phase HCT116 cells, CDK7 inhibition reduced S130 phosphorylation and further increased or stabilized the binding of p21 to these complexes. In agreement with previous reports, such increased stoichiometry [10], [80], and/or stability [9], of p21 binding is likely sufficient to explain the impaired T172 phosphorylation and activity of CDK4 complexes and their refractoriness to *in vitro* activation by CAK. Mutations of the S130 residue and *in vitro* phosphorylation indeed suggested that this phosphorylation can reduce the stoichiometry or stability of p21 binding to CDK4 complexes, resulting in increased T172 phosphorylation of p21-bound CDK4. Interestingly, T187 phosphorylation of p27 also facilitates its release from cyclin E-CDK2 complexes [77]. At later time points, inhibition of S130 phosphorylation likely also explains the increased accumulation of p21 in response to CDK7 inhibition, in agreement with previous studies [61]–[63]. This too might suffice to prevent phosphorylation and activation of CDK4 complexes, as observed in response to proteasome inhibition by MG132.

There are no crystallographic or biophysical data to explain how cyclin D-CDK4 complexes might possibly accommodate more than one p21 molecule, and thus how this might be affected by S130 phosphorylation. p21 is an intrinsically unstructured, flexible protein containing two cyclin- and one CDK-binding domain [64], [71]. In the case of cyclin D1-CDK4, the interaction of p21 with CDK4 and the resulting inhibition of its activity have been reported to depend mainly on both the N-terminal Cy1 cyclin-binding motif and the K CDK-binding site [64], [81]. However, the involvement of the C-terminal Cy2 cyclin-binding site is unclear. Whereas a Cy2 peptide is unable to affect the cyclin D1-CDK4-p21 interaction, a longer peptide containing residues 127 to 164 of p21 (thus encompassing both the Cy2 and S130) efficiently blocks the p21 interaction [64]. Therefore, further experiments should investigate if the S130 residue is directly involved in the interaction of p21 in such a way that the phosphorylation of this site would alter the p21 inhibitory activity.

The p21 S130 residue can be phosphorylated by cyclin E-CDK2 [61], [62] and also by CDK6 activated by the viral cyclin K [79]. Using 2D gel separations, we show that the *in vitro* phosphorylation of p21 by cyclin E/A-CDK2 occurs mainly at S130 but also at S98 (a residue reported to be phosphorylated by ASK1 and JNK1 but not CDKs [82]), and we show for the first time that cyclin D-CDK4/6 also readily phosphorylate S130. Like p27 in cyclin E-CDK2 complexes [12], p21 is thus both a potential inhibitor and a substrate of cyclin D-CDK4/6 complexes. Interestingly, S130 was more efficiently phosphorylated by the different CDK complexes when p21 was bound to cyclin D-CDK4. This, together with two other observations, suggests that p21 is not phosphorylated merely via an autonomous “intra-complex” mechanism. First, binding to inactive CDK4 complexes similarly increased the S130 phosphorylation of p21 in transfected CHO cells (Figure 4C). Second, S130 phosphorylation did not appear after incubation of p21-cyclin D3-CDK4 complexes with high ATP concentrations but no other kinase (Figure 3B). p21 as a subunit of the CDK4 complex would thus be phosphorylated, and hence regulated, by other active CDK4 and CDK2 complexes. As for CDK2-dependent phosphorylation of p21 on S130 *in vitro* [61], deletion of the C-terminal Cy2 cyclin-binding site much reduced p21 phosphorylation in HCT116 cells (Figure S11), suggesting that in cyclin D/CDK4-bound p21 the interaction with other active cyclin-CDK complexes to phosphorylate S130 is mediated by the Cy2 site. In intact HCT116 and CHO cells, the involvement of both CDK4 and CDK2 in S130 phosphorylation was further established by its inhibition in response to increased p21 expression, inhibition of CDK4/6 by PD0332991, and/or inhibition of CDKs by roscovitine and CR8 (which do not directly inhibit CDK4 activity). CR8 did not inhibit CDK7 activity in these experiments and thus most likely acted by inhibiting CDK2. In all those situations, much reduced T172 phosphorylation and activity of p21-bound CDK4 were associated with the inhibition of p21 S130 phosphorylation. To our knowledge, this is the first indication that CDK2 might control CDK4 activation.

Our data suggest that, depending on their activities during cell cycle progression, either CDK4 or CDK2 complexes can exert positive feedbacks to reduce the negative impact of p21 on CDK4 activation (Figure 9). This could further amplify CDK4 activation and subsequently lead to increased degradation of p21, which in turn facilitates CDK2 activation. Our data indicate that p21 inhibitory activity can be deactivated inside the inhibited cyclin D-CDK4 complex by other active CDK4 complexes. If this can be verified, theoretical conditions would be met to generate a bistable biochemical system (see e.g. [83] for a mathematical discussion of the analogous deactivation of p27 by cyclin E-CDK2). In such a switch-like biochemical module, binary (all-or-none) activation of CDK4 could occur, and sustain its permissiveness to activation despite reduced mitogenic stimulation, once the threshold barrier imposed by the p21 inhibitory potential has been overcome [84]. The positive “reverse” feedback pathways from CDK4 and CDK2 to CDK4 activation proposed here for the first time would introduce the notion that not only the pRb-E2F switch [5], but also its trigger, might be endowed with bistable properties that would contribute to explaining the irreversibility of the cell cycle commitment at the R point. This mechanism (or similar ones in p21-independent CDK4 activation as observed in cAMP-dependent cell cycle of thyrocytes [39], [85]) can explain the exceptional robustness of the correlation between CDK4 T172 phosphorylation, pRb phosphorylation and subsequent S-phase entry that we have observed in all the cell models and regulations we studied.

A future challenge will be to identify the mechanisms that engage the positive feedback amplifying system that we suggest here for the activation of CDK4 complexes stabilized by p21, which represent the bulk of CDK4 complexes in G1 phase. Mitogenic signal transduction kinases might directly phosphorylate the p21 S130 residue [86]. However, this possibility is not supported by the disappearance of S130 phosphorylation associated with CDK inhibition. Alternatively, less stable but very active cyclin D-CDK4 complexes exist without binding to p21 or p27 [31], [58]–[60], the activation of which would depend on yet unknown non-CDK7 activating kinases as suggested here. Our *in vitro* experiments (Figure S6) raise the interesting possibility that a minimal amount of such hyperactive p21/p27-unbound CDK4 complexes might initiate the autocatalytic activation of a significant proportion of p21-bound CDK4 complexes through S130 phosphorylation (acting *as kindling to ignite the pyre*) (Figure 9).

### CDK4 phosphorylation as a convergent target of cancer therapies

The corollary of the central position of T172-phosphorylation of CDK4 in the cell cycle decision module is its responsiveness to various drugs or treatments used or envisaged in cancer therapy. CDK4 phosphorylation is a central node integrating oncogenic signaling cascades, as its abrogation requires combined inhibition of the Ras/MEK/ERK and mTOR pathways [37]. But amazingly, it is also inhibited by various CDK inhibitors that were not suspected to affect CDK4 activity, including roscovitine (Seliciclib), and drugs that increase p21 levels, including proteasome inhibitors, such as Bortezomib. In p53-proficient cells, genotoxic therapeutic drugs and treatments, which induce large amounts of p21, are thus also expected to inhibit CDK4 phosphorylation. The resulting inhibition of CDK4 activity may halt cell proliferation in pRb-proficient tumors. On the other hand, p21-dependent inhibition of CDK4 activation might also explain the stronger resistance of pRb-proficient cells to the apoptotic effect of these different treatments, which might be exploited to protect normal cells and hence allow more aggressive treatments of pRb-deficient tumors [29], [87]. Finally, the novel positive feedback pathways identified here from CDK4 and CDK2 to facilitate CDK4 activation may explain the relative resistance to receptor or cell signaling kinase inhibitory drugs of cancer cells that have intrinsically strong CDK4 activity (e.g. due to frequent *CDKN2A* deletion or *Cdk4* amplification) and/or CDK2 activity (e.g. due to deregulation of cyclin E). This would provide a rationale for treatments that combine inhibitory drugs targeting oncogenic signaling pathways and CDKs, as suggested recently [88].

## Materials and Methods

### Cell culture, BrdU incorporation, and transfection

These procedures were preformed exactly as described [33], [53]. p21-null HCT116 cells were provided by Vogelstein Lab (Baltimore). After starvation without FBS for 3 days, HCT116 cells were growth-stimulated with 10% FBS. Inhibitors were dissolved in DMSO and used at the following final concentrations: 20 µM roscovitine (Sigma), 10 µM 1-NMPP1 (Santa Cruz Biotechnology), 250 nM or 1 µM PD0332991, as indicated in figures legends (Selleck Chemicals), 0.5 µM CR8 (kind gift from Laurent Meijer, or Sigma), 10 µM MG132 (Calbiochem). Vehicle (0.2% DMSO) was added in the controls. DNA replicating cells were identified by 30-min incubation with BrdU. The incorporation of BrdU was detected by immunofluorescence: BrdU-labeled nuclei were counted as described [36]. CHO and HCT116 cells were transfected in 9-cm Petri dishes for 48 h or 24 h, respectively, using Fugene (Roche Diagnostics) with 6 µg of pcDNA6 vector encoding HA-tagged CDK4 and Xpress-cyclin D3, and 6 µg or 0.6 µg pE vectors encoding human p21 and its mutants. All constructs were verified by sequencing.

### Lentivirus production, cell infection and selection

HEK293T cells were transfected by calcium phosphate for 6 h with a mix of vectors: pSPAX2, pMD2G (Trono Lab), pDG2iV5puro encoding N-terminus 3×HA-tagged human p21 or its mutants, or pLVX-rtTA3 (TetON) or Tight-puro (Clontech) encoding Flag tagged CDK6 wt, CDK6 S178P mutant or cyclin D3. Multi-cistronic pDG2iV5puro is a modified version of the pLenti6 V5/DEST vector (Invitrogen). It contains an expression cassette under the control of a Tet-responsive element allowing doxycycline-inducible expression of the gene of interest and an SV40 promoter controlling rtTA3 transactivator expression followed by an IRES and a puromycin-resistance marker. Transfected HEK293T supernatant was collected after 2 days, clarified, filtered, and concentrated on Amicon filter (Millipore). HCT116 cells were infected for 24 h by adding to the culture medium a volume of lentiviruses corresponding to 100 µg of p24 capsid protein per 50×10^3^ cells (titrated by ELISA according to the manufacturer's protocol, INNOTEST HIV Antigen mAb, Innogenetics). Infected cells were selected by antibiotic resistance with a final concentration of 0.4 µg/ml of puromycin (Invivogene).

### Immunoprecipitation, pRb-kinase assay, and 2D gel electrophoresis

(Co-)Immunoprecipitations were performed as described [31], [89] using the following antibodies : monoclonal antibodies against cyclin D1 (DCS-11) or cyclin D3 (DCS-28) (all from Neomarkers); monoclonal antibody against HA epitope (F7) and polyclonal antibodies against p21 (C-19), CDK6 (C-21), CDK7 (C-19) and cyclin H (C-18) (all from Santa Cruz Biotechnology); monoclonal anti-Flag (M2) (Sigma).

To assay pRb-kinase activity, immunoprecipitated protein complexes were incubated with ATP and a recombinant pRb fragment (QED or Sigma). The mixture was then separated by SDS-PAGE and western blotted to detect T826 phosphorylation of the pRb fragment and co-immunoprecipitated proteins [31], [89]. For 2D gel electrophoresis, separations were performed exactly as described [31], [33], [41], and the immunoprecipitations were denatured in a buffer containing 7 M urea and 2 M thiourea. Proteins were separated by isoelectric focusing on immobilized linear pH gradient strips (pH 3 to 10) and then separated by SDS-PAGE and immunoblotted.

### *In vitro* phosphorylation

Complexes containing CDK4 or CDK6 from HCT116 cells, p21 from CHO cells transfected with p21 vector alone, or with cyclin D3 or cyclin D1-Flag and CDK4 vectors, or HA-p21 from stably infected HCT116 K7AS by lentiviruses expressing inducible 3×HA-p21 wt or its mutants were immunoprecipitated and used as substrate for *in vitro* phosphorylation. When indicated, immunoprecipitates were first dephosphorylated with λ-phosphatase as described [33]. The immunoprecipitates were washed three times with NP40 buffer (150 mM NaCl, 50 mM Tris-HCl, pH 7.5, 0.5% Nonidet P-40, 50 mM NaF, 1 mM sodium orthovanadate, 1 mM DTT, protease inhibitors (pefablock and leupeptin) and once with CAK buffer (80 mM β-glycerophosphate, pH 7.3, 15 mM MgCl_2_, 20 mM EGTA, 5 mM DTT). The beads were resuspended in 50 µl of CAK buffer containing protease and phosphatase inhibitors and 2 mM ATP and incubated at 30°C for 1 h with or without 1 µg of recombinant kinases: CDK2-cyclin E1, CDK2-cyclin A2 and CDK4-cyclin D3 (Carna Biosciences); cyclin H-CDK7-MAT1 (Upstate); CDK4-cyclin D1 and CDK6-cyclin D1 (ProQinase) .

### CAK activity assay

Cyclin D3-CDK4 complexes from transfected CHO cells were immunoprecipitated and dephosphorylated with λ-phosphatase as described [33]. These complexes were used as a substrate for activation by CAK complexes immunoprecipitated from HCT116 K7AS cells. Their *in vitro* activation was then assessed by their pRb-kinase activity [36]. Dephosphorylated cyclin D3-CDK4 complexes were washed three times with NP-40 lysis buffer and then three times with CAK buffer. The beads were mixed in 30 µl of CAK buffer containing protease and phosphatase inhibitors with cyclin H-CDK7 complexes (coimmunoprecipitated using the C-18 cyclin H antibody) from K7AS cells either treated or not treated with FBS or CR8. After addition of 1 mM ATP, the suspensions were incubated at 30°C for 30 min. After three washes in CAK buffer and three washes in pRb kinase buffer, the immunoprecipitated proteins were assayed for pRb kinase activity as described above.

### Immunoblot analysis

SDS-PAGE separations of equal amounts of whole cell extract proteins [89], and immunoprecipitates were immunodetected using the following antibodies: monoclonal antibodies against cyclin D1 (DCS-6), cyclin D3 (DCS-22), p27 (DCS-72), p21 (DCS60) (all from Neomarkers); monoclonal antibody anti-cyclin E (HE12) (Pierce Biotechnology); monoclonal anti-cyclin A E43.2 (kind gift from Tim Hunt); anti-total pRb monoclonal antibody (#554136, BD Pharmingen); polyclonal anti-phospho-pRb antibodies (T826 and T821) (Biosource-Invitrogen); polyclonal antibodies anti-phospho-pRb (S807/811), anti-phospho-CDK2 (T160), anti-phospho-histone H3 (S10) (all from Cell Signaling Technology); monoclonal antibody against HA epitope (F7), CDK7 (C-4) and MAT1 (F-6), polyclonal antibodies against CDK4 (C-22), CDK6 (C-21), CDK2 (M-2), cyclin H (C-18), phospho-CDK7 (T170), XPD (TFIIH p80) (all from Santa Cruz Biotechnology); monoclonal antibodies against total RNA Polymerase II (8WG16), phospho-Ser2 (H5) or phospho-Ser5 (H14) of RNA Polymerase II (all from Covance). Secondary antibodies were either coupled to horseradish peroxidase (Amersham Biosciences) for detection by enhanced chemiluminescence (Western Lightning, Perkin-Elmer) or to DyLight 680 and 800 (Pierce Biotechnology) for infrared fluorescence detection using the Odyssey scanner (LI-COR).

## Supporting Information

Figure S1 (Related to Figure 1). Characterization of DNA synthesis (A), cell cycle protein levels (B) and CDK4 phosphorylation during cell cycle progression in HCT116 K7AS cells. HCT116 WT (A) or K7AS (A–C) cells were synchronized by serum deprivation for two days and re-stimulated with 10% fetal bovine serum (FBS) for different times. (A) DNA synthesis was evaluated from duplicate dishes by counting the percentage of cells having incorporated BrdU during the last 30 min of stimulation. (B) Western blotting analysis was performed with the indicated antibodies from whole-cell lysates. *, pRb hypophosphorylated band; **, pRb hyperphosphorylated bands. (C) Cell lysates were immunoprecipitated (IP) with anti-cyclin D1 (D1) or anti-cyclin D3 (D3) antibodies and separated by 2D gel electrophoresis followed by CDK4 detection. Arrows, T172-phosphorylated form of CDK4. Different exposures are shown for the different time points to better visualize the proportion of the CDK4 phosphorylated form irrespective of the relative amount of cyclin D-CDK4 complexes. In K7AS HCT116 (K7AS), DNA synthesis started to increase between 6 and 8 h after stimulation and peaked at 12–16 h (Figure S1A). As readout of CDK4 and CDK6 activity, T826 phosphorylation of pRb was first observed to increase at 3 h and peaked at 16 h (Figure S1B). Cyclin D1 and cyclin D3 expression was first seen to increase at 2 h. Whereas cyclin D1 accumulation peaked at 6 h, cyclin D3 continued to accumulate during S and G2 phases until 24 h. CDK4 and CDK6 expression was much less modulated (Figure S1B). Interestingly, the phosphorylation of cyclin D1-bound CDK4 appeared at 2–3 h into G1 phase, whereas the phosphorylation of cyclin D3-bound CDK4 was already detected in serum-deprived cells and further increased much later at 12 h and subsequent time points, when most cells were in S-G2 phases (Figure S1C). This suggests that CDK4 complexed to cyclin D1 and cyclin D3 might have partially different roles in the different cell cycle phases. The activating T160 phosphorylation of CDK2 was observed to increase at 4–6 h, along with an increased accumulation of cyclin E and a migration shift of this protein (likely associated with its CDK2-dependent phosphorylation [90]). This coincided with the partial disappearance of p21 and p27, which reappeared at later time points (20–24 h) (Figure S1B).
(TIF)

Figure S2 (Related to Figure 1). Specific inhibition of CDK7 by 1-NMPP1 prevents T826 phosphorylation of pRb and T160 phosphorylation of CDK2 while increasing p21 accumulation (A). Specific inhibition of CDK7 also prevents the activating phosphorylation (B) and pRb-kinase activity of CDK6 (C). WT (A) and K7AS (A–C) HCT116 cells were stimulated (+) or not stimulated (−) with fetal bovine serum (FBS) for the indicated times in the absence (−) or presence (+) of 1-NMPP1. (A) Western blotting analysis with the indicated antibodies from whole-cell lysates. (B,C) Cell lysates (analyzed in Figure 1B–1D) were immunoprecipitated (IP) with anti-cyclin D1 (D1) or anti-cyclin D3 (D3) and separated by 2D gel electrophoresis followed by CDK6 immunodetection (B), or were immunoprecipitated with anti-CDK6 antibody, assayed for pRb-kinase activity, separated by SDS-PAGE, and immunoblotted with the indicated antibodies (C). Arrows, position of the T177-phosphorylated form of CDK6.
(TIF)

Figure S3 Unlike cyclin D3-CDK6, CDK4 complexes from CDK7-inhibited cells are refractory to *in vitro* phosphorylation by CAK. HCT116 K7AS cells were stimulated (+) or not stimulated (−) with fetal bovine serum (FBS) for 5 h in the absence (−) or presence (+) of 1-NMPP1. Cell lysates were immunoprecipitated (IP) with anti-cyclin D1 (D1), anti-cyclin D3 (D3) or anti-p21 antibodies and incubated with ATP in the presence (+) or absence (−) of recombinant cyclin H-CDK7-MAT1 complex (CAK). The complexes were then separated by 2D gel electrophoresis and immunodetected with a mixture of anti-CDK4 and anti-CDK6 antibodies. In the inset, as a positive control of CAK activity in the same experiment, immunoprecipitated (D3 IP) cyclin D3-CDK4 complexes from CHO cells transfected with plasmids encoding cyclin D3 and CDK4-HA were pretreated or not with λ-phosphatase (λ PPase) and then incubated with ATP with or without CAK, before 2D gel electrophoresis and CDK4 immunodetection. Arrows indicate the position of T172/T177-phosphorylated form of CDK4/6. If the impaired activation of CDK4 and CDK6 complexes in CDK7-inhibited K7AS cells was due only to absence of activating phosphorylation, these complexes should remain phosphorylatable by CAK *in vitro*, as observed here using cyclin D3-CDK4 complexes produced in CHO cells (inset). By contrast, only cyclin D3-CDK6, but neither cyclin D3-CDK4, cyclin D1-CDK4 nor cyclin D1-CDK6, was phosphorylated by CAK from 1-NMPP1-treated cells. This refractoriness to CAK activation of cyclin D3-CDK4 from CDK7-inhibited cells might have been due to its increased association with p21 (Figure 1C). Indeed, p21-bound CDK4 and CDK6 (which in part were associated with cyclin D3) from CDK7-inhibited cells were also refractory to phosphorylation by CAK. Consistently, the observation that only cyclin D3-CDK6 but not cyclin D3-CDK4 from CDK7-inhibited cells could be activated by CAK is likely explained by the weaker binding, and hence relative resistance, of cyclin D3-CDK6 complexes to p21 [91]. Unexpectedly, irrespective of the cell treatment by serum or 1-NMPP1, none of the cyclin D1 complexes could be re-activated by CAK in these experiments. Others have also observed that only cyclin D3-CDK6 but neither cyclin D1-CDK6 nor cyclin D1-CDK4 could be activated by CAK *in vitro* [32]. As T-loop phosphorylation of CDK7 might be more important for its recognition of S/T-P motifs (as found in CDK4 and most non-CDK substrates of CDK7) [92], we also verified that the recombinant CDK7 was largely phosphorylated at T170 using 2D-gel electrophoresis combined with a T170-phosphospecific antibody (data not shown).
(TIF)

Figure S4 (Related to Figure 3A). (A) As in Figure 3A, HCT116 K7AS cells were stimulated (+) or not stimulated (−) with fetal bovine serum (FBS) for 5 h in the absence (−) or presence (+) of 1-NMPP1. Cell lysates were immunoprecipitated with anti-p21 antibody and separated by 2D gel electrophoresis followed by p21 immunodetection. This strong exposure allows observation of the doubly phosphorylated form of p21. (B) As in Figure 2, K7AS cells were stimulated with FBS for 5 h and 1-NMPP1 was added or not added for 1 h. Cell lysates were immunoprecipitated with anti-cyclin D1 antibody (D1 IP) and separated by 2D gel electrophoresis followed by p21 immunodetection. (C) Kinetics of the appearance of phosphorylated forms of p21 and T172 phosphorylation of p21-bound CDK4 during cell cycle progression. HCT116 K7AS cells were stimulated or not stimulated with FBS for the indicated times. Cell lysates were immunoprecipitated (IP) with anti p21 antibody and separated by 2D gel electrophoresis followed by CDK4 and p21 detection. Black arrows, T172-phosphorylated form of CDK4. Different exposures are shown for the different time points to better visualize the proportion of the different forms of p21 and CDK4 irrespective of the relative amounts of p21 and p21-bound CDK4 complexes. Colored arrows indicate phosphorylated forms of p21 identified by their characteristic migration and mutagenesis as analyzed in Figure S5 and Figure 3B, 3C.
(TIF)

Figure S5 (Related to Figure 3B, 3C). Identification of the phosphorylated forms of p21. CHO cells were transfected with plasmids encoding wild-type p21 (WT) or the indicated mutants of p21, alone (A) or together with plasmids encoding cyclin D3 and CDK4-HA (B). Cell lysates were immunoprecipitated (IP) with anti-cyclin D3 (D3) (B) or anti-p21 antibodies (A) and incubated with the indicated recombinant kinases and ATP. The proteins were separated by 2D gel electrophoresis followed by p21 immunodetection. Right panel in (B), superimposition of 2D gel profiles of WT p21 (colorized in green) and the indicated mutants of p21 (colorized in red) after phosphorylation by cyclin A-CDK2. Arrows indicate the main phosphorylated forms of p21.
(TIF)

Figure S6 (Related to Figure 3B, 3C). Binding to cyclin D1-CDK4 presents p21 for S130 phosphorylation by cyclin D1-CDK4 and cyclin D1-CDK6. CHO cells were transfected with plasmids encoding p21 alone (left column) or together with plasmids encoding cyclin D1-Flag and CDK4-HA (right column). Cell lysates were immunoprecipitated (IP) with anti-p21 (left) or anti-Flag antibodies (right) and incubated with ATP and the indicated recombinant kinases. The proteins were separated by 2D gel electrophoresis followed by p21 immunodetection. Arrows indicate the phosphorylated forms of p21.
(TIF)

Figure S7 Proteasomal inhibition increases p21 levels and mimics the effects of CDK7 inhibition on phosphorylations of pRb and CDK2 (A), pRb kinase activity (B), and T172 phosphorylation of CDK4 and S130 phosphorylation of p21 (C). HCT116 K7AS cells were restimulated or not restimulated with fetal bovine serum (+/− FBS) during 5 or 16 h in the continuous absence (−) or presence (+) of MG132. (A) Western blotting analysis was performed with the indicated antibodies from whole-cell lysates. (B,C) Lysates from cells treated for 5 h were immunoprecipitated (IP) with anti-p21 or anti-cyclin D1 (D1) antibodies and assayed for their pRb-kinase activity, separated by SDS-PAGE and immunoblotted with the indicated antibodies (B), or were separated by 2D gel electrophoresis followed by CDK4 and p21 immunodetection (C).
(TIF)

Figure S8 (Related to Figure 5). (A) In the context of K domain T57D mutation, S130D phosphomimetic mutation of p21 weakens p21 binding to CDK4 and increases phosphorylation of the remaining CDK4 bound to p21. Stably infected HCT116 K7AS cells for Tet-On inducible 3×HA-p21 wt or indicated mutants were treated with doxycycline (1 µg/ml) for 16 h prior to cell restimulation with fetal bovine serum (FBS) for 16 h in the continuous presence of doxycycline. Cell lysates were immunoprecipitated (IP) with anti-HA antibody and separated by 2D gel electrophoresis followed by simultaneous immunodetection of ectopic 3×HA-p21 and 3×HA-p21-bound endogenous CDK4 using a mixture of anti-CDK4 and p21 antibodies. (B) Phosphorylation of p21 by cyclin A-CDK2 potentially modifies the interaction of p21 with cyclin D-CDK4 to increase the accessibility of CDK4 T172 to CAK. Stably infected HCT116 K7AS cells allowing doxycycline-inducible HA-p21 wt or T57D mutant expression were serum-deprived for 48 h and treated with doxycycline (1 µg/ml) during 16 h. Cell lysates were immunoprecipitated (IP) with anti-HA (HA) antibody and incubated first with ATP with or without recombinant cyclin A2-CDK2 and then with ATP with or without recombinant cyclin H-CDK7-MAT1 (CAK). The proteins were separated by 2D gel electrophoresis followed by simultaneous immunodetection of ectopic 3×HA-p21 and 3×HA-p21-bound endogenous CDK4 using a mixture of anti-CDK4 and p21 antibodies. Right panels in (A,B) are CDK4 enlargements with adjustment of exposure times to facilitate comparison of the proportion of the phosphorylated form (arrows). Colored arrows in (B) indicate phosphorylated forms of p21. The experiment in Figure S8A suggested that S130 phosphorylation of p21 may somehow weaken its interaction with cyclin D-CDK4 complexes, allowing CDK4 phosphorylation by CAK or another kinase. As no previous study has reported that S130 phosphorylation could be mimicked by the S130D mutation (unlike the homologous T187D mutation of p27), we wanted to evaluate whether *in vitro* phosphorylation of p21-cyclin-CDK4 complexes by CDK2 might more efficiently affect them and the capacity of p21-bound CDK4 to be phosphorylated by CAK. As shown in Figure S8B, codetection of p21 and CDK4 after *in vitro* phosphorylation by cyclin A2-CDK2 and/or CAK revealed that (i) cyclin A2-CDK2 phosphorylated p21 at S130, S98 and another unidentified site (lane3; as previously observed in Figure 3B). Of note, CAK also phosphorylated p21 at another unidentified site (not S130, T57 or S98 as demonstrated by different migration; lane 2); (ii) phosphorylation of wt p21 by cyclin A2-CDK2 did not affect CDK4 co-immunoprecipitation and did not appreciably increase subsequent phosphorylation of p21-bound CDK4 by CAK (lane 4); (iii) however, in the T57D mutation context, phosphorylation by cyclin A2-CDK2 much reduced CDK4 interaction with p21 (lane 7), allowing complete phosphorylation by CAK of the remaining p21-bound CDK4 (lane 8).
(TIF)

Figure S9 (Related to Figure 6). (A) Effect of roscovitine, 1-NMPP1 and CR8 on RNA polymerase II phosphorylation. HCT116 K7AS cells were stimulated (+) or not stimulated (−) with fetal bovine serum (FBS) for 5 h in the absence or presence of the following inhibitors: roscovitine (rosco), 1-NMPP1, CR8 or a combination of 1-NMPP1 and CR8. Western blotting analysis was performed with the indicated antibodies from whole-cell lysates. To evaluate the impact of R-roscovitine and CR8 on CDK7 in Figure 6 experiments, we compared their effect to specific inhibition of CDK7 by 1-NMPP1 on phosphorylations of C-terminal domain (CTD) of the large subunit of RNA polymerase II. Previous studies in K7AS HCT116 cells have shown that CTD S5 phosphorylation is performed by both CDK7 and CDK9, whereas CTD S2 might be an exclusive CDK9 substrate [53], [93]. As previously shown [53], CDK7 inhibition by 1-NMPP1 was insufficient to affect CTD S5 and S2 phosphorylations. By contrast, roscovitine inhibited both phosphorylations, whereas CR8 inhibited only, but completely, S2 phosphorylation, confirming its inhibitory impact on CDK9 [94]. Interestingly, 1-NMPP1 did abrogate S5 phosphorylation in the presence of CR8 (A). This confirmed that inhibition of CTD S5 phosphorylation requires combined inhibition of both CDK7 and CDK9, and also implies that CR8 did not affect CDK7 activity. Overall, we concluded that, in addition to strong CDK2 inhibition, roscovitine reduced CDK7 and CDK9 activities, whereas CR8 inhibited CDK2 and CDK9 but not CDK7. Effect of roscovitine on phosphorylations of p21 and CDK4 thus most likely resulted from inhibition of both CDK2 and CDK7, whereas CR8 acted only through CDK2 inhibition. (B) The effect of CDK2 inhibition by CR8 on CDK4 phosphorylation is delayed compared to its abrupt disappearance resulting from CDK7 inhibition. HCT116 K7AS cells were stimulated with fetal bovine serum (FBS) for 5 h and roscovitine (rosco), CR8 or 1-NMPP1 were added or not added for 1 h. Cell lysates were immunoprecipitated (IP) with anti-cyclin D1 (D1), anti-cyclin D3 (D3) or anti-p21 antibodies and separated by 2D gel electrophoresis followed by CDK4 immunodetection.
(TIF)

Figure S10 (Related to Figure 6C). Serum stimulation and CDK2 inhibition by CR8 in HCT116 K7AS cells has little effect on the composition and activity of CAK (cyclin H-CDK7-MAT1) complexes (A) and phosphorylation profile of CDK7 (B). (A,B) K7AS cells were stimulated or not stimulated (cont) with fetal bovine serum (FBS) for 5 h in the absence or presence of CR8 as in Figure 6C. (A) The activity of co-immunoprecipitated cyclin H-CDK7 complexes (cyc H IP) from these cells was evaluated on CDK4 complexes used a substrate. In this assay, these cyclin H-CDK7 complexes were mixed and incubated with ATP and inactive (dephosphorylated by λ-phosphatase) cyclin D3-CDK4-HA complexes produced and immunoprecipitated from transfected CHO cells [33]. The resulting activation of the cyclin D3-CDK4-HA complexes was then assayed by their pRb-kinase activity. The mixtures were separated by SDS-PAGE and immunoblotted. We detected cyclin H (cyc H) and CDK7, T170-phosphorylated CDK7, MAT1 and XPD co-immunoprecipitated by the cyclin H antibody from K7AS cells, and the presence of the substrate, i.e. cyclin D3-CDK4-HA complexes from CHO cells (CDK4-HA), and its *in vitro* activation reflected by the T826 phosphorylation of the pRb fragment (pRb-kinase). (B) Phosphorylation profiles of CDK7. Same cell lysates as in (A) were immunoprecipitated (IP) with anti-CDK7 or anti-cyclin H (cyc H) and separated by 2D gel electrophoresis followed by immunodetection by antibodies directed against CDK7 or T170-phosphorylated CDK7 (P-CDK7 (T170)). As deduced from computed isoelectric points [40] and detection by the T170-phosphospecific antibody, arrowheads and arrows indicate singly and doubly phosphorylated CDK7 forms, respectively. The doubly phosphorylated forms are most likely phosphorylated at both T170 and S164 [51].
(TIF)

Figure S11 Phosphorylations of p21 and p21-bound CDK4 depend on the Cy2 cyclin-binding motif of p21. Stably infected HCT116 K7AS cells for Tet-On inducible 3×HA-p21 wt or ΔCy2 (aa 1 to 154) mutant were treated with doxycycline (1 µg/ml) for 16 h prior cell restimulation with fetal bovine serum (FBS) for 16 h in the continuous presence of doxycycline. Cell lysates were immunoprecipitated (IP) with anti-HA antibody and separated by 2D gel electrophoresis followed by simultaneous immunodetection of ectopic 3×HA-p21 and 3×HA-p21-bound endogenous CDK4 using a mixture of anti-CDK4 and p21 antibodies. Black arrow, T172-phosphorylated CDK4. Colored arrows indicate phosphorylated forms of p21. Black arrowheads indicate the unphosphorylated form of wt and ΔCy2 p21. Of note, deletion of the 10 C-terminal aminoacids (including the Cy2 motif) much modifies the isoelectric point of p21 (computed isoelectric points of 3×HA-p21 and its ΔCy2 mutant are 7.63 and 5.61, respectively). This explains why the phosphorylated forms are much less separated in the ΔCy2 mutant, which also precludes the formal identification of these forms (the form indicated by a blue arrow is putatively phosphorylated on S98).
(TIF)
