# Supplementary figures and images for: CDK4 T172 Phosphorylation Is Central in a CDK7-Dependent Bidirectional CDK4/CDK2 Interplay Mediated by p21 Phosphorylation at the Restriction Point
Source: PLoS Genet. 2013 May 30;9(5):e1003546. doi: 10.1371/journal.pgen.1003546 (PMC3667761; doi:10.1371/journal.pgen.1003546)

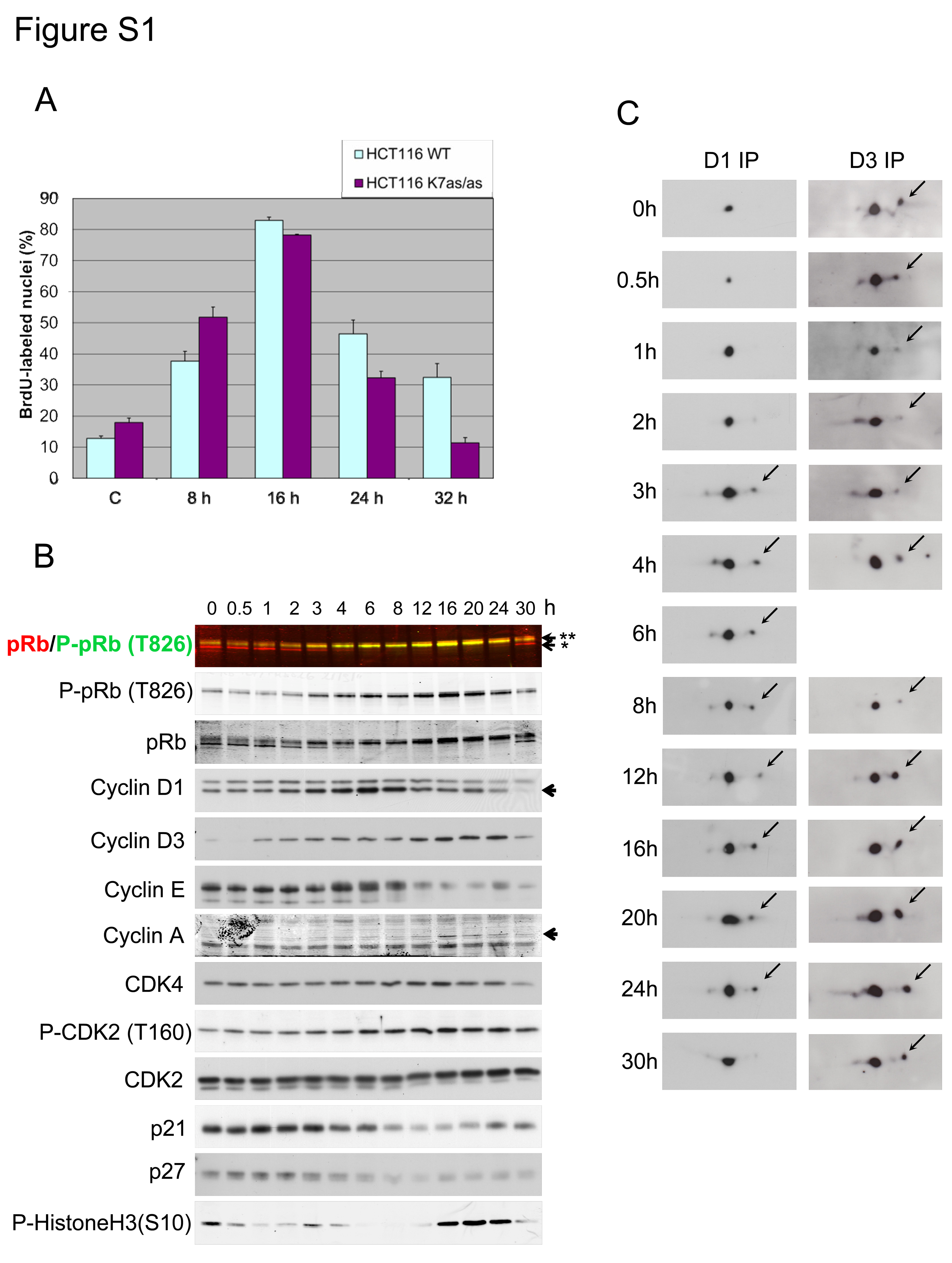

Supplement: Figure S1 — (Related to Figure 1). Characterization of DNA synthesis (A), cell cycle protein levels (B) and CDK4 phosphorylation during cell cycle progression in HCT116 K7AS cells. HCT116 WT (A) or K7AS (A–C) cells were synchronized by serum deprivation for two days and re-stimulated with 10% fetal bovine serum (FBS) for different times. (A) DNA synthesis was evaluated from duplicate dishes by counting the percentage of cells having incorporated BrdU during the last 30 min of stimulation. (B) Western blotting analysis was performed with the indicated antibodies from whole-cell lysates. *, pRb hypophosphorylated band; **, pRb hyperphosphorylated bands. (C) Cell lysates were immunoprecipitated (IP) with anti-cyclin D1 (D1) or anti-cyclin D3 (D3) antibodies and separated by 2D gel electrophoresis followed by CDK4 detection. Arrows, T172-phosphorylated form of CDK4. Different exposures are shown for the different time points to better visualize the proportion of the CDK4 phosphorylated form irrespective of the relative amount of cyclin D-CDK4 complexes. In K7AS HCT116 (K7AS), DNA synthesis started to increase between 6 and 8 h after stimulation and peaked at 12–16 h (Figure S1A). As readout of CDK4 and CDK6 activity, T826 phosphorylation of pRb was first observed to increase at 3 h and peaked at 16 h (Figure S1B). Cyclin D1 and cyclin D3 expression was first seen to increase at 2 h. Whereas cyclin D1 accumulation peaked at 6 h, cyclin D3 continued to accumulate during S and G2 phases until 24 h. CDK4 and CDK6 expression was much less modulated (Figure S1B). Interestingly, the phosphorylation of cyclin D1-bound CDK4 appeared at 2–3 h into G1 phase, whereas the phosphorylation of cyclin D3-bound CDK4 was already detected in serum-deprived cells and further increased much later at 12 h and subsequent time points, when most cells were in S-G2 phases (Figure S1C). This suggests that CDK4 complexed to cyclin D1 and cyclin D3 might have partially different roles in the different cell cycl [file pgen.1003546.s001.tif]

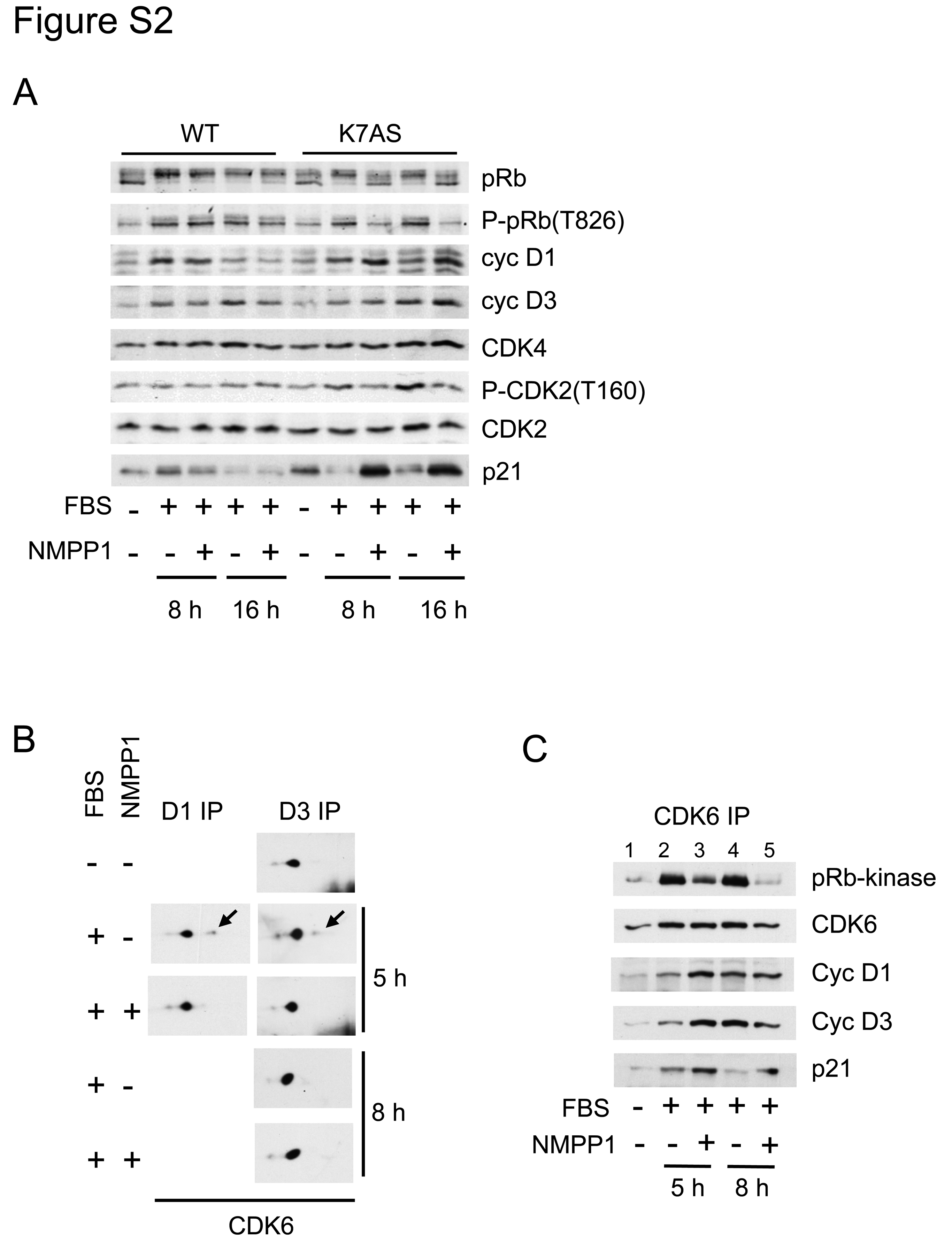

Supplement: Figure S2 — (Related to Figure 1). Specific inhibition of CDK7 by 1-NMPP1 prevents T826 phosphorylation of pRb and T160 phosphorylation of CDK2 while increasing p21 accumulation (A). Specific inhibition of CDK7 also prevents the activating phosphorylation (B) and pRb-kinase activity of CDK6 (C). WT (A) and K7AS (A–C) HCT116 cells were stimulated (+) or not stimulated (−) with fetal bovine serum (FBS) for the indicated times in the absence (−) or presence (+) of 1-NMPP1. (A) Western blotting analysis with the indicated antibodies from whole-cell lysates. (B,C) Cell lysates (analyzed in Figure 1B–1D) were immunoprecipitated (IP) with anti-cyclin D1 (D1) or anti-cyclin D3 (D3) and separated by 2D gel electrophoresis followed by CDK6 immunodetection (B), or were immunoprecipitated with anti-CDK6 antibody, assayed for pRb-kinase activity, separated by SDS-PAGE, and immunoblotted with the indicated antibodies (C). Arrows, position of the T177-phosphorylated form of CDK6. (TIF) [file pgen.1003546.s002.tif]

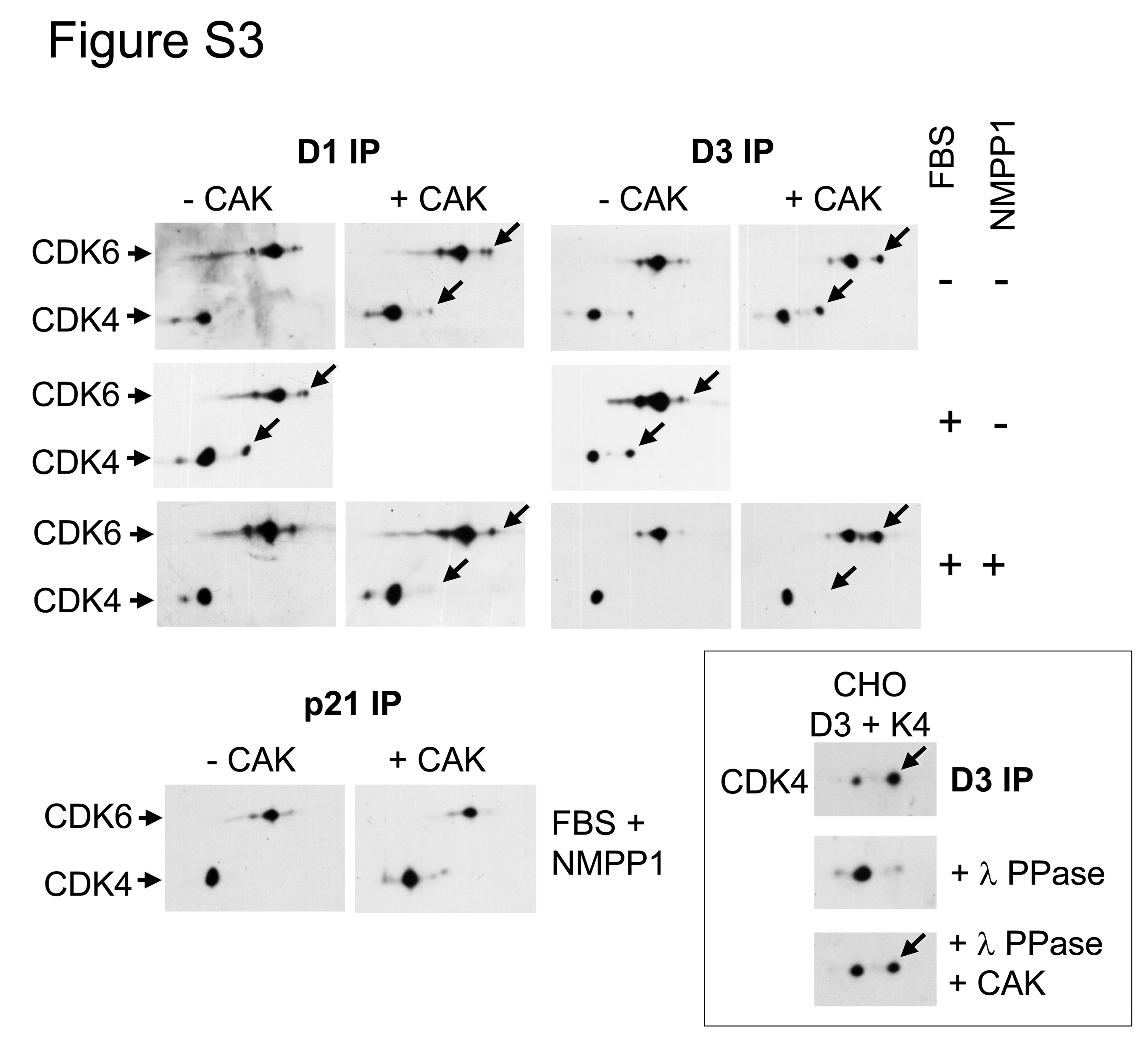

Supplement: Figure S3 — Unlike cyclin D3-CDK6, CDK4 complexes from CDK7-inhibited cells are refractory to in vitro phosphorylation by CAK. HCT116 K7AS cells were stimulated (+) or not stimulated (−) with fetal bovine serum (FBS) for 5 h in the absence (−) or presence (+) of 1-NMPP1. Cell lysates were immunoprecipitated (IP) with anti-cyclin D1 (D1), anti-cyclin D3 (D3) or anti-p21 antibodies and incubated with ATP in the presence (+) or absence (−) of recombinant cyclin H-CDK7-MAT1 complex (CAK). The complexes were then separated by 2D gel electrophoresis and immunodetected with a mixture of anti-CDK4 and anti-CDK6 antibodies. In the inset, as a positive control of CAK activity in the same experiment, immunoprecipitated (D3 IP) cyclin D3-CDK4 complexes from CHO cells transfected with plasmids encoding cyclin D3 and CDK4-HA were pretreated or not with λ-phosphatase (λ PPase) and then incubated with ATP with or without CAK, before 2D gel electrophoresis and CDK4 immunodetection. Arrows indicate the position of T172/T177-phosphorylated form of CDK4/6. If the impaired activation of CDK4 and CDK6 complexes in CDK7-inhibited K7AS cells was due only to absence of activating phosphorylation, these complexes should remain phosphorylatable by CAK in vitro, as observed here using cyclin D3-CDK4 complexes produced in CHO cells (inset). By contrast, only cyclin D3-CDK6, but neither cyclin D3-CDK4, cyclin D1-CDK4 nor cyclin D1-CDK6, was phosphorylated by CAK from 1-NMPP1-treated cells. This refractoriness to CAK activation of cyclin D3-CDK4 from CDK7-inhibited cells might have been due to its increased association with p21 (Figure 1C). Indeed, p21-bound CDK4 and CDK6 (which in part were associated with cyclin D3) from CDK7-inhibited cells were also refractory to phosphorylation by CAK. Consistently, the observation that only cyclin D3-CDK6 but not cyclin D3-CDK4 from CDK7-inhibited cells could be activated by CAK is likely explained by the weaker binding, and hence relative resistance, of cyclin D3-CDK6 [file pgen.1003546.s003.tif]

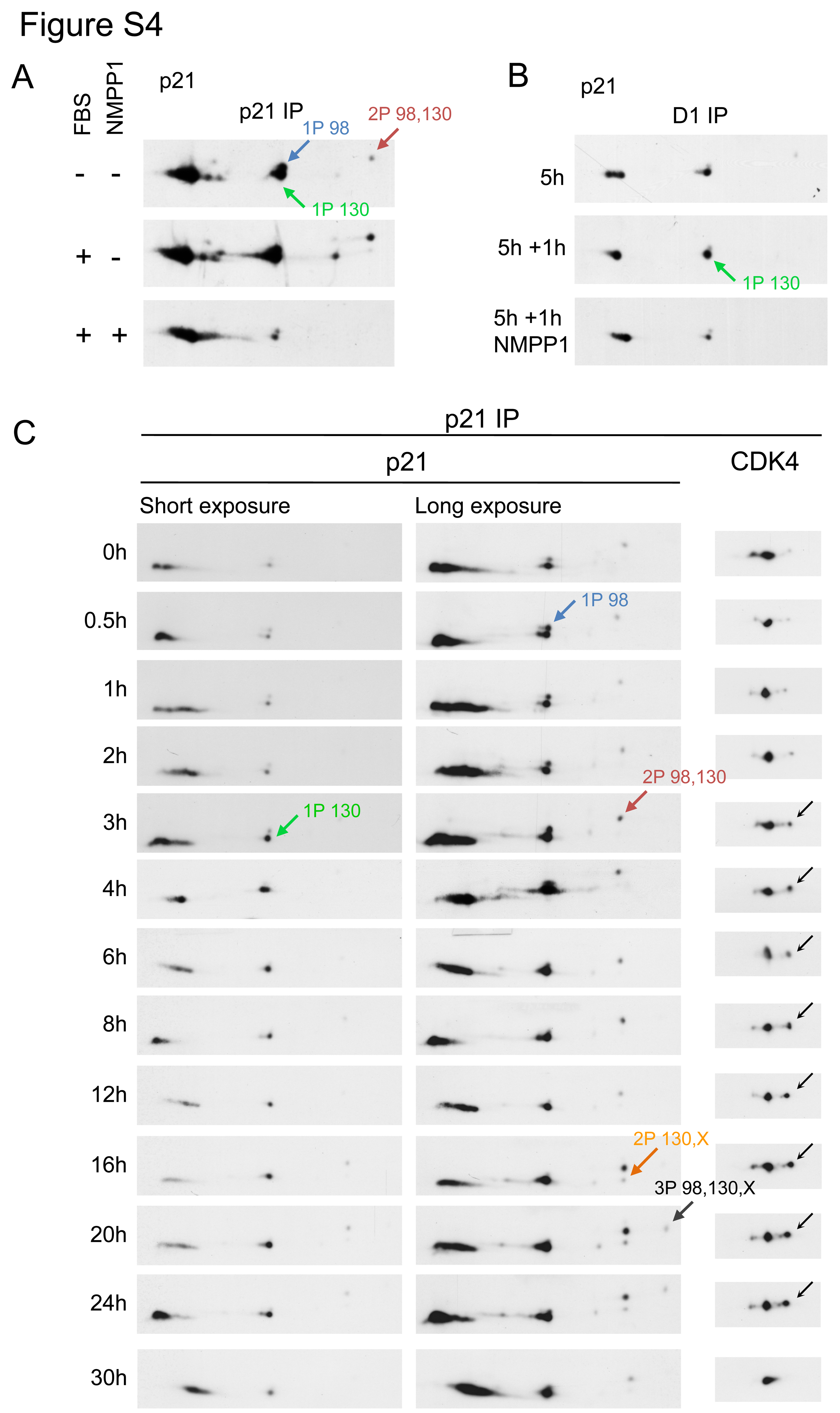

Supplement: Figure S4 — (Related to Figure 3A). (A) As in Figure 3A, HCT116 K7AS cells were stimulated (+) or not stimulated (−) with fetal bovine serum (FBS) for 5 h in the absence (−) or presence (+) of 1-NMPP1. Cell lysates were immunoprecipitated with anti-p21 antibody and separated by 2D gel electrophoresis followed by p21 immunodetection. This strong exposure allows observation of the doubly phosphorylated form of p21. (B) As in Figure 2, K7AS cells were stimulated with FBS for 5 h and 1-NMPP1 was added or not added for 1 h. Cell lysates were immunoprecipitated with anti-cyclin D1 antibody (D1 IP) and separated by 2D gel electrophoresis followed by p21 immunodetection. (C) Kinetics of the appearance of phosphorylated forms of p21 and T172 phosphorylation of p21-bound CDK4 during cell cycle progression. HCT116 K7AS cells were stimulated or not stimulated with FBS for the indicated times. Cell lysates were immunoprecipitated (IP) with anti p21 antibody and separated by 2D gel electrophoresis followed by CDK4 and p21 detection. Black arrows, T172-phosphorylated form of CDK4. Different exposures are shown for the different time points to better visualize the proportion of the different forms of p21 and CDK4 irrespective of the relative amounts of p21 and p21-bound CDK4 complexes. Colored arrows indicate phosphorylated forms of p21 identified by their characteristic migration and mutagenesis as analyzed in Figure S5 and Figure 3B, 3C. (TIF) [file pgen.1003546.s004.tif]

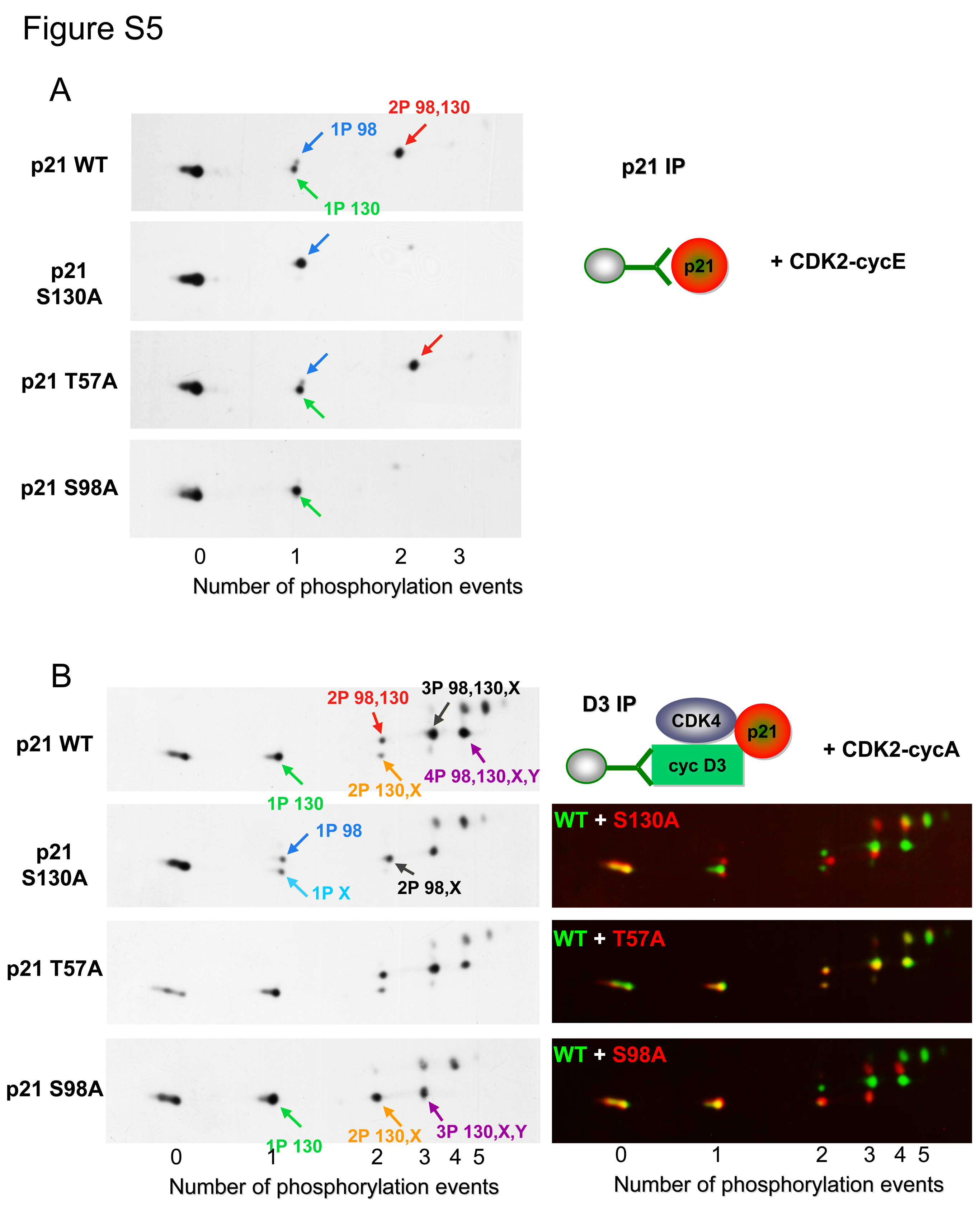

Supplement: Figure S5 — (Related to Figure 3B, 3C). Identification of the phosphorylated forms of p21. CHO cells were transfected with plasmids encoding wild-type p21 (WT) or the indicated mutants of p21, alone (A) or together with plasmids encoding cyclin D3 and CDK4-HA (B). Cell lysates were immunoprecipitated (IP) with anti-cyclin D3 (D3) (B) or anti-p21 antibodies (A) and incubated with the indicated recombinant kinases and ATP. The proteins were separated by 2D gel electrophoresis followed by p21 immunodetection. Right panel in (B), superimposition of 2D gel profiles of WT p21 (colorized in green) and the indicated mutants of p21 (colorized in red) after phosphorylation by cyclin A-CDK2. Arrows indicate the main phosphorylated forms of p21. (TIF) [file pgen.1003546.s005.tif]

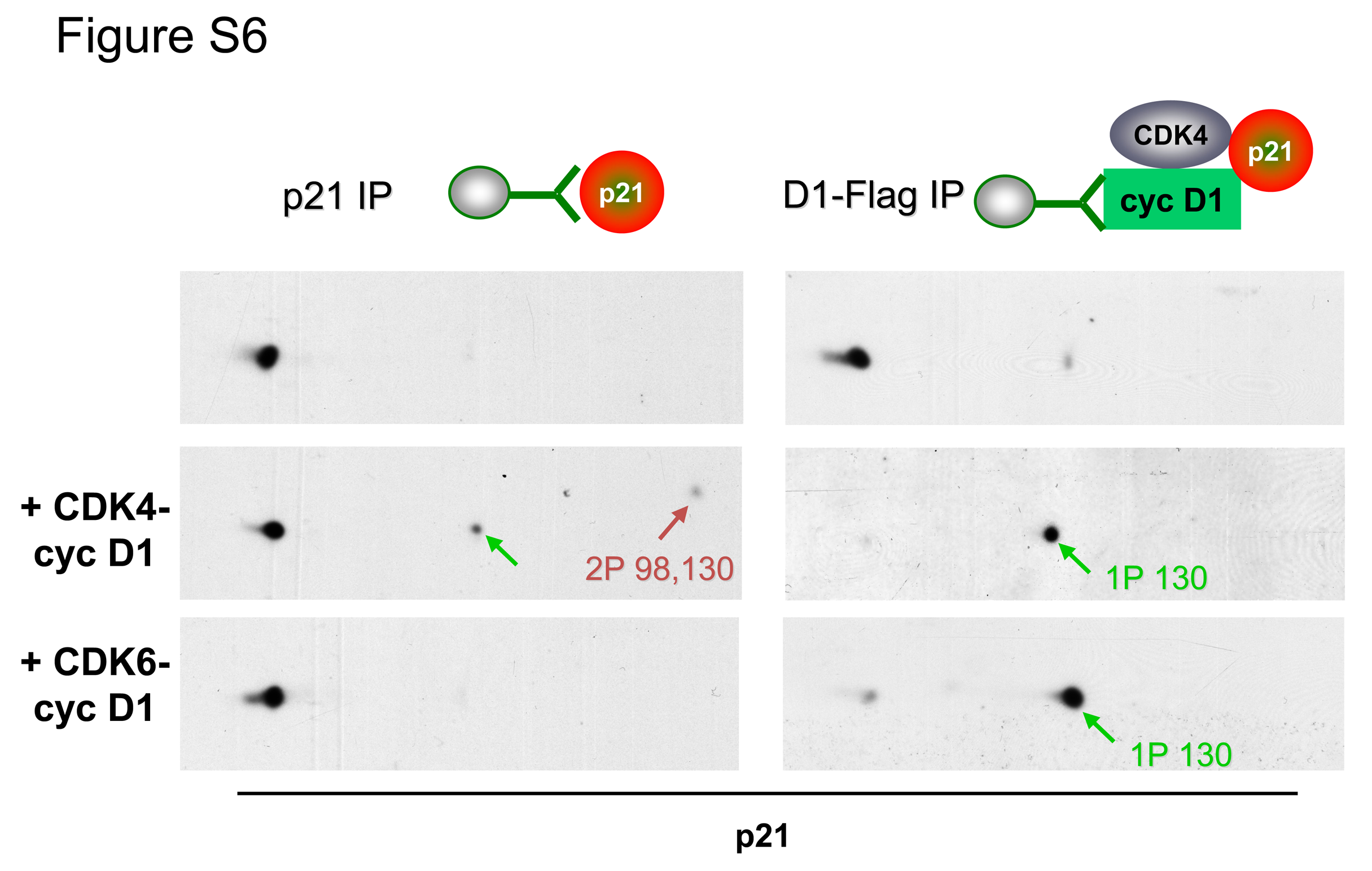

Supplement: Figure S6 — (Related to Figure 3B, 3C). Binding to cyclin D1-CDK4 presents p21 for S130 phosphorylation by cyclin D1-CDK4 and cyclin D1-CDK6. CHO cells were transfected with plasmids encoding p21 alone (left column) or together with plasmids encoding cyclin D1-Flag and CDK4-HA (right column). Cell lysates were immunoprecipitated (IP) with anti-p21 (left) or anti-Flag antibodies (right) and incubated with ATP and the indicated recombinant kinases. The proteins were separated by 2D gel electrophoresis followed by p21 immunodetection. Arrows indicate the phosphorylated forms of p21. (TIF) [file pgen.1003546.s006.tif]

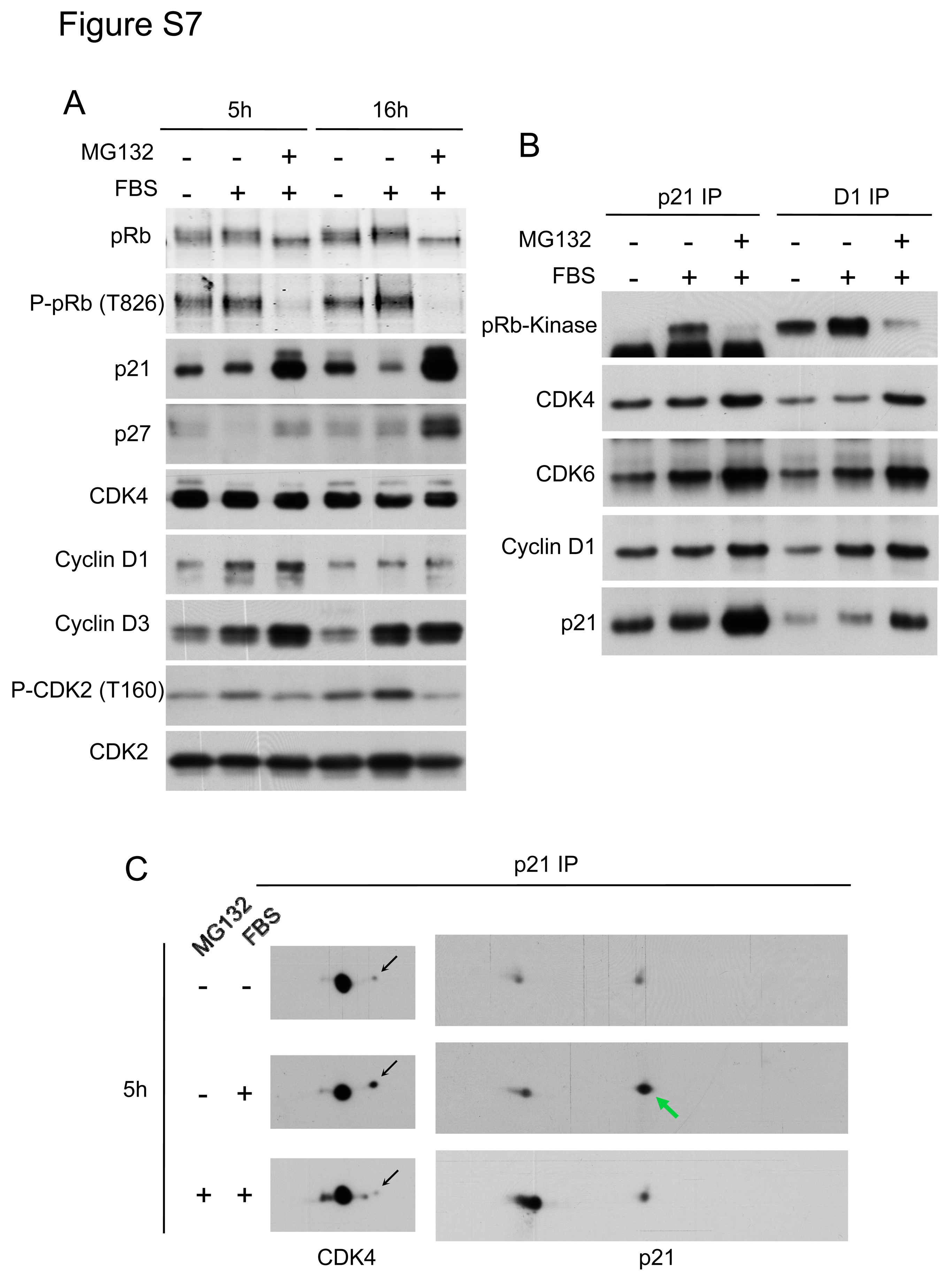

Supplement: Figure S7 — Proteasomal inhibition increases p21 levels and mimics the effects of CDK7 inhibition on phosphorylations of pRb and CDK2 (A), pRb kinase activity (B), and T172 phosphorylation of CDK4 and S130 phosphorylation of p21 (C). HCT116 K7AS cells were restimulated or not restimulated with fetal bovine serum (+/− FBS) during 5 or 16 h in the continuous absence (−) or presence (+) of MG132. (A) Western blotting analysis was performed with the indicated antibodies from whole-cell lysates. (B,C) Lysates from cells treated for 5 h were immunoprecipitated (IP) with anti-p21 or anti-cyclin D1 (D1) antibodies and assayed for their pRb-kinase activity, separated by SDS-PAGE and immunoblotted with the indicated antibodies (B), or were separated by 2D gel electrophoresis followed by CDK4 and p21 immunodetection (C). (TIF) [file pgen.1003546.s007.tif]

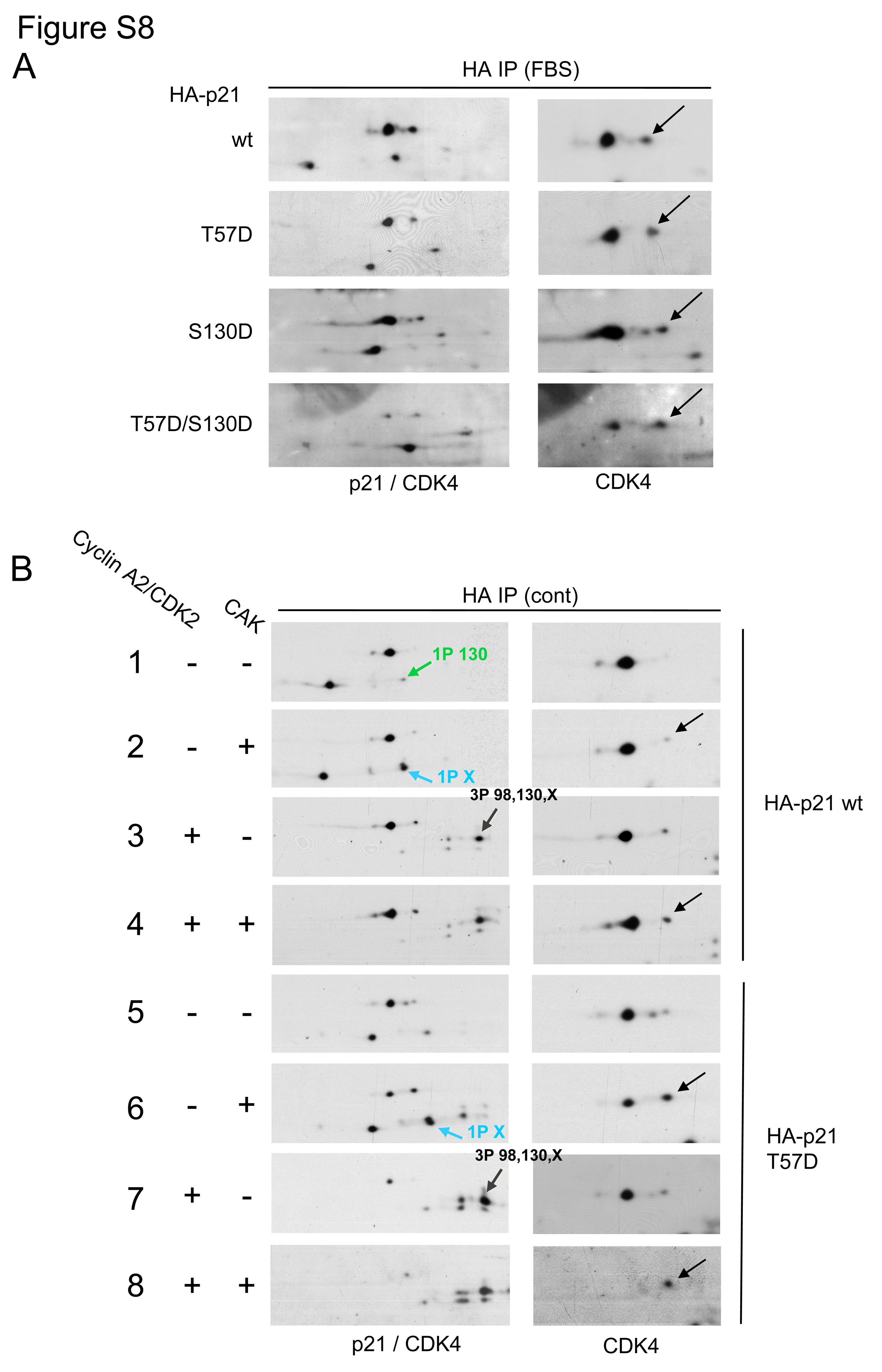

Supplement: Figure S8 — (Related to Figure 5). (A) In the context of K domain T57D mutation, S130D phosphomimetic mutation of p21 weakens p21 binding to CDK4 and increases phosphorylation of the remaining CDK4 bound to p21. Stably infected HCT116 K7AS cells for Tet-On inducible 3×HA-p21 wt or indicated mutants were treated with doxycycline (1 µg/ml) for 16 h prior to cell restimulation with fetal bovine serum (FBS) for 16 h in the continuous presence of doxycycline. Cell lysates were immunoprecipitated (IP) with anti-HA antibody and separated by 2D gel electrophoresis followed by simultaneous immunodetection of ectopic 3×HA-p21 and 3×HA-p21-bound endogenous CDK4 using a mixture of anti-CDK4 and p21 antibodies. (B) Phosphorylation of p21 by cyclin A-CDK2 potentially modifies the interaction of p21 with cyclin D-CDK4 to increase the accessibility of CDK4 T172 to CAK. Stably infected HCT116 K7AS cells allowing doxycycline-inducible HA-p21 wt or T57D mutant expression were serum-deprived for 48 h and treated with doxycycline (1 µg/ml) during 16 h. Cell lysates were immunoprecipitated (IP) with anti-HA (HA) antibody and incubated first with ATP with or without recombinant cyclin A2-CDK2 and then with ATP with or without recombinant cyclin H-CDK7-MAT1 (CAK). The proteins were separated by 2D gel electrophoresis followed by simultaneous immunodetection of ectopic 3×HA-p21 and 3×HA-p21-bound endogenous CDK4 using a mixture of anti-CDK4 and p21 antibodies. Right panels in (A,B) are CDK4 enlargements with adjustment of exposure times to facilitate comparison of the proportion of the phosphorylated form (arrows). Colored arrows in (B) indicate phosphorylated forms of p21. The experiment in Figure S8A suggested that S130 phosphorylation of p21 may somehow weaken its interaction with cyclin D-CDK4 complexes, allowing CDK4 phosphorylation by CAK or another kinase. As no previous study has reported that S130 phosphorylation could be mimicked by the S130D mutation (unlike the homologous T187D mutation of [file pgen.1003546.s008.tif]

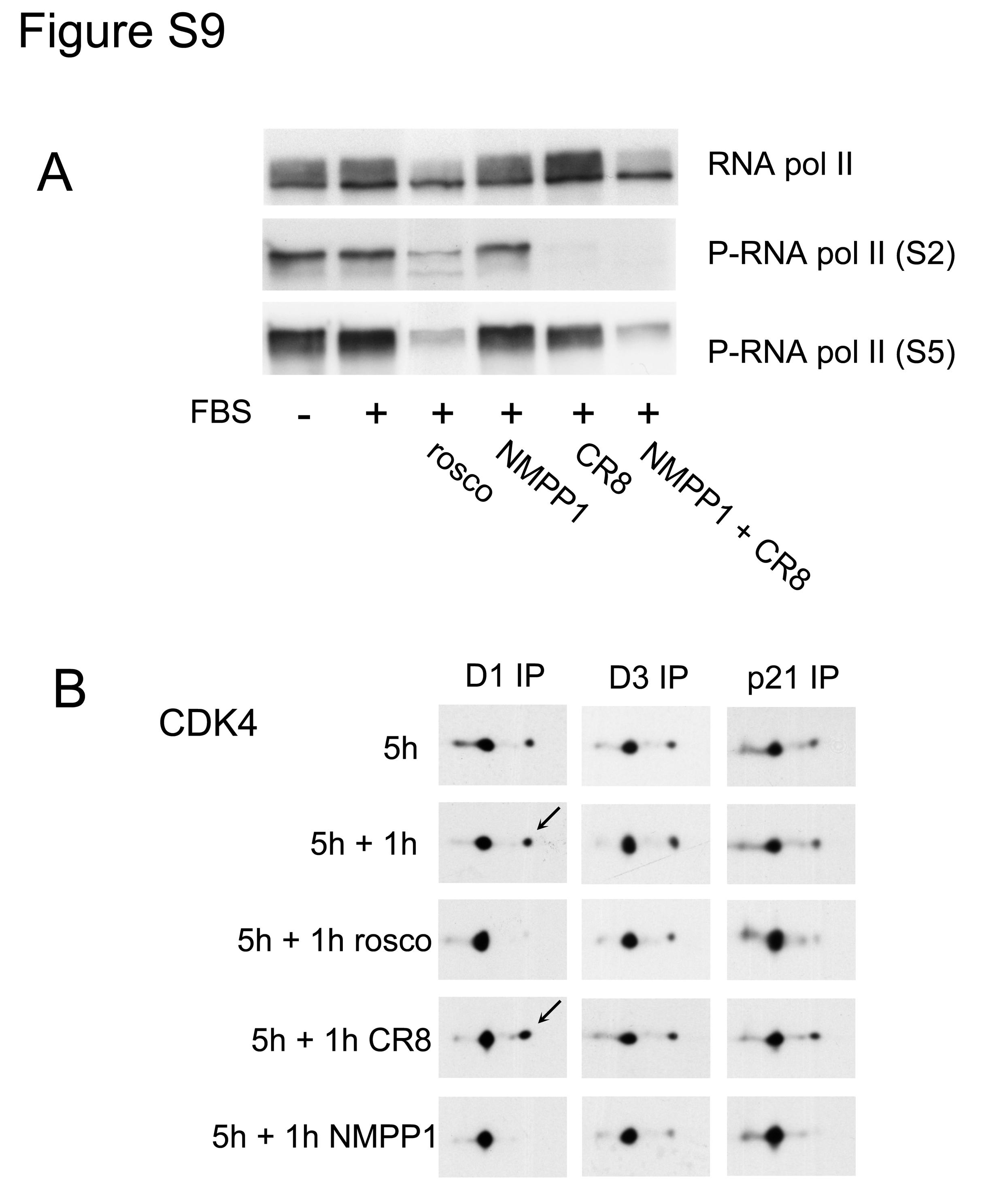

Supplement: Figure S9 — (Related to Figure 6). (A) Effect of roscovitine, 1-NMPP1 and CR8 on RNA polymerase II phosphorylation. HCT116 K7AS cells were stimulated (+) or not stimulated (−) with fetal bovine serum (FBS) for 5 h in the absence or presence of the following inhibitors: roscovitine (rosco), 1-NMPP1, CR8 or a combination of 1-NMPP1 and CR8. Western blotting analysis was performed with the indicated antibodies from whole-cell lysates. To evaluate the impact of R-roscovitine and CR8 on CDK7 in Figure 6 experiments, we compared their effect to specific inhibition of CDK7 by 1-NMPP1 on phosphorylations of C-terminal domain (CTD) of the large subunit of RNA polymerase II. Previous studies in K7AS HCT116 cells have shown that CTD S5 phosphorylation is performed by both CDK7 and CDK9, whereas CTD S2 might be an exclusive CDK9 substrate [53], [93]. As previously shown [53], CDK7 inhibition by 1-NMPP1 was insufficient to affect CTD S5 and S2 phosphorylations. By contrast, roscovitine inhibited both phosphorylations, whereas CR8 inhibited only, but completely, S2 phosphorylation, confirming its inhibitory impact on CDK9 [94]. Interestingly, 1-NMPP1 did abrogate S5 phosphorylation in the presence of CR8 (A). This confirmed that inhibition of CTD S5 phosphorylation requires combined inhibition of both CDK7 and CDK9, and also implies that CR8 did not affect CDK7 activity. Overall, we concluded that, in addition to strong CDK2 inhibition, roscovitine reduced CDK7 and CDK9 activities, whereas CR8 inhibited CDK2 and CDK9 but not CDK7. Effect of roscovitine on phosphorylations of p21 and CDK4 thus most likely resulted from inhibition of both CDK2 and CDK7, whereas CR8 acted only through CDK2 inhibition. (B) The effect of CDK2 inhibition by CR8 on CDK4 phosphorylation is delayed compared to its abrupt disappearance resulting from CDK7 inhibition. HCT116 K7AS cells were stimulated with fetal bovine serum (FBS) for 5 h and roscovitine (rosco), CR8 or 1-NMPP1 were added or not added for 1 h. Cell lys [file pgen.1003546.s009.tif]

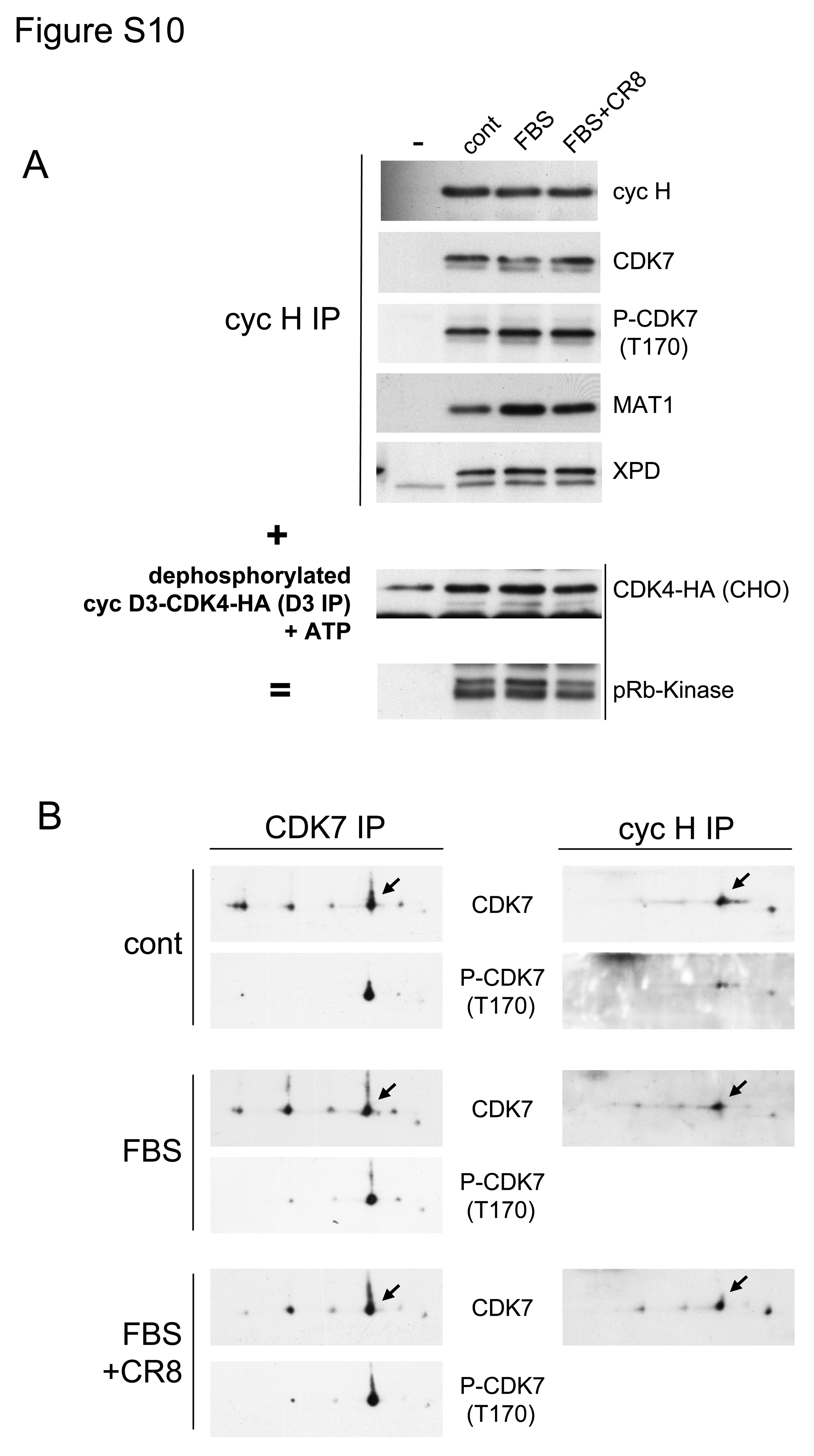

Supplement: Figure S10 — (Related to Figure 6C). Serum stimulation and CDK2 inhibition by CR8 in HCT116 K7AS cells has little effect on the composition and activity of CAK (cyclin H-CDK7-MAT1) complexes (A) and phosphorylation profile of CDK7 (B). (A,B) K7AS cells were stimulated or not stimulated (cont) with fetal bovine serum (FBS) for 5 h in the absence or presence of CR8 as in Figure 6C. (A) The activity of co-immunoprecipitated cyclin H-CDK7 complexes (cyc H IP) from these cells was evaluated on CDK4 complexes used a substrate. In this assay, these cyclin H-CDK7 complexes were mixed and incubated with ATP and inactive (dephosphorylated by λ-phosphatase) cyclin D3-CDK4-HA complexes produced and immunoprecipitated from transfected CHO cells [33]. The resulting activation of the cyclin D3-CDK4-HA complexes was then assayed by their pRb-kinase activity. The mixtures were separated by SDS-PAGE and immunoblotted. We detected cyclin H (cyc H) and CDK7, T170-phosphorylated CDK7, MAT1 and XPD co-immunoprecipitated by the cyclin H antibody from K7AS cells, and the presence of the substrate, i.e. cyclin D3-CDK4-HA complexes from CHO cells (CDK4-HA), and its in vitro activation reflected by the T826 phosphorylation of the pRb fragment (pRb-kinase). (B) Phosphorylation profiles of CDK7. Same cell lysates as in (A) were immunoprecipitated (IP) with anti-CDK7 or anti-cyclin H (cyc H) and separated by 2D gel electrophoresis followed by immunodetection by antibodies directed against CDK7 or T170-phosphorylated CDK7 (P-CDK7 (T170)). As deduced from computed isoelectric points [40] and detection by the T170-phosphospecific antibody, arrowheads and arrows indicate singly and doubly phosphorylated CDK7 forms, respectively. The doubly phosphorylated forms are most likely phosphorylated at both T170 and S164 [51]. (TIF) [file pgen.1003546.s010.tif]

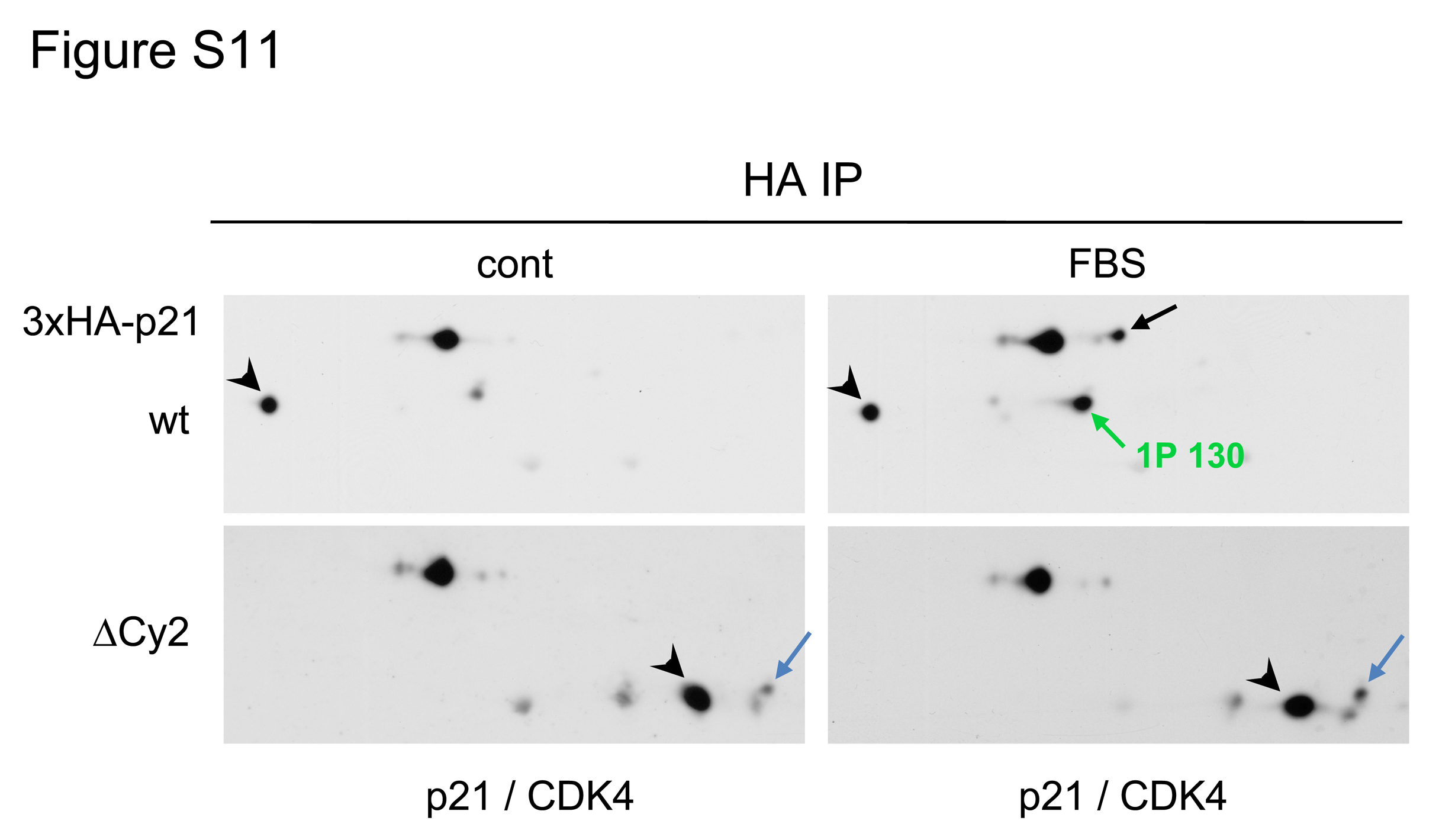

Supplement: Figure S11 — Phosphorylations of p21 and p21-bound CDK4 depend on the Cy2 cyclin-binding motif of p21. Stably infected HCT116 K7AS cells for Tet-On inducible 3×HA-p21 wt or ΔCy2 (aa 1 to 154) mutant were treated with doxycycline (1 µg/ml) for 16 h prior cell restimulation with fetal bovine serum (FBS) for 16 h in the continuous presence of doxycycline. Cell lysates were immunoprecipitated (IP) with anti-HA antibody and separated by 2D gel electrophoresis followed by simultaneous immunodetection of ectopic 3×HA-p21 and 3×HA-p21-bound endogenous CDK4 using a mixture of anti-CDK4 and p21 antibodies. Black arrow, T172-phosphorylated CDK4. Colored arrows indicate phosphorylated forms of p21. Black arrowheads indicate the unphosphorylated form of wt and ΔCy2 p21. Of note, deletion of the 10 C-terminal aminoacids (including the Cy2 motif) much modifies the isoelectric point of p21 (computed isoelectric points of 3×HA-p21 and its ΔCy2 mutant are 7.63 and 5.61, respectively). This explains why the phosphorylated forms are much less separated in the ΔCy2 mutant, which also precludes the formal identification of these forms (the form indicated by a blue arrow is putatively phosphorylated on S98). (TIF) [file pgen.1003546.s011.tif]
